# Acrylonitrile‐Mediated Nascent RNA Sequencing for Transcriptome‐Wide Profiling of Cellular RNA Dynamics

**DOI:** 10.1002/advs.201900997

**Published:** 2020-03-05

**Authors:** Yuqi Chen, Fan Wu, Zonggui Chen, Zhiyong He, Qi Wei, Weiwu Zeng, Kun Chen, Feng Xiao, Yushu Yuan, Xiaocheng Weng, Yu Zhou, Xiang Zhou

**Affiliations:** ^1^ College of Chemistry and Molecular Sciences Wuhan University Wuhan 430072 P. R. China; ^2^ The Institute for Advanced Studies Wuhan University Wuhan 430072 P. R. China; ^3^ State Key Laboratory of Virology College of Life Sciences Wuhan University Wuhan 430072 P. R. China

**Keywords:** acrylonitrile, G‐quadruplex, nascent RNA sequencing, RNA dynamics, transcriptome‐wide profiling, uridine‐to‐cytidine conversion

## Abstract

RNA sequencing has greatly facilitated gene expression studies but is weak in studying temporal RNA dynamics; this issue can be addressed by analyzing nascent RNAs. A famous method for nascent RNA analysis is metabolic labeling with noncanonic nucleoside followed by affinity purification, however, purification processes can always introduce biases into data analysis. Here, a chemical method for nascent RNA sequencing that avoids affinity purification based on acrylonitrile‐mediated uridine‐to‐cytidine (U‐to‐C) conversion (AMUC‐seq) via 4‐thiouridine (s^4^U) cyanoethylation is presented. This method converts s^4^U base‐pairing with guanine through the nucleophilic addition of s^4^U to acrylonitrile. The high reaction efficiency permits AMUC‐seq directly and efficiently to recover nascent RNA information from total RNAs. AMUC‐seq is validated by being used to detect mRNA half‐lives and investigating the direct gene targets of a G‐quadruplex stabilizer, which can be regarded as potential anticancer drug, in human cells. Thousands of direct gene targets of this drug are verified (these genes are significantly enriched in cancer such as *SRC* and *HRAS*). AMUC‐seq also confirms G‐quadruplex stabilization that impacts RNA polyadenylation. These results show AMUC‐seq is qualified for the study of temporal RNA dynamics, and it can be a promising strategy to study the therapeutic mechanism of transcription‐modulating drugs.

## Graphical abstract

4‐thiouridine (s^4^U) cyanoethylation converts in the nascent RNA to cytidine in a base‐pairing manner, representing a promising strategy for enrichment‐free nascent RNA detection that further benefits transcriptome‐wide analysis of temporal RNA dynamics.

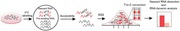

## 1 Introduction

RNA sequencing (RNA‐seq) is a revolutionary tool for transcriptome profiling in a given biological sample that allows us to look at the dynamic changes of gene expression against different conditions or stimuli.^[^ ^1^ ^]^ Understanding transcript dynamics is essential for characterizing the underlying mechanisms of different regulatory processes, which further benefits the discovery of disease markers and potential drug targets. However, traditional RNA‐seq technology is generally performed at the steady‐state level of cellular RNAs, changes in RNA transcription and decay rates cannot be easily distinguished. In addition, it provides poor resolution of the temporal information of RNA kinetics. Cells always require relatively long hours of stimuli to achieve detectable differential expression upon RNA‐seq, and multiple secondary signaling events could be promoted and contribute to the observed changes within that time, which confuses the underlying regulatory mechanisms.^[^ ^2^ ^]^

To address these issues, recently, new strategies for analyzing newly synthesized (nascent) RNA instead of total cellular RNA have been developed.^[^ ^3^ ^]^ Nascent RNA profiling can reveal the temporal information of gene expression changes. Metabolic labeling and downstream purification of metabolically labeled nascent RNA followed by RNA sequencing has become a famous approach to study nascent RNA behaviors. For example, global run‐on sequencing (GRO‐seq)^[^ ^4^ ^]^ uses 5Br‐UTP to label nascent RNA and then nascent RNAs containing 5Br‐U are immune‐precipitated with an anti‐BrU antibody.^[^ ^4^ ^]^ 4‐thiouridine (s^4^U) and 5‐ethynyl uridine (5EU) can also be used for nascent RNA labeling and then act as chemical handles for the separation of labeled RNA via thio/ethynyl‐specific biotinylation.^[^ ^5^, ^6^ ^]^ However, these techniques require large amounts of input sample. And the conventional purification assay for isolating metabolically labeled nascent RNAs is laborious, a certain amount of contamination can be induced during purification processes due to the non‐specific binding. Sometimes RNA purification suffers from limited efficiency and tends to enrich transcripts that contain high uridine content (Figure S1, Supporting Information). These issues can result in experimental bias unless additional experiments and/or computational processing is employed for data normalization.^[^ ^7^, ^8^ ^]^

Recently, an innovative enrichment‐free strategy for nascent RNA detection has been presented, adding a new dimension for the study of RNA dynamics.^[^ ^9^ ^]^ This strategy directly distinguished nascent RNA from total RNA population in single‐base resolution by marking the mapping reads of nascent RNA with introduced base mutations. Briefly, cells are exposed to a thiol‐labeled nucleoside (s^4^U or s^6^G), resulting in rapid uptake and incorporation into the newly synthesized RNA, which can be then isolated and treated with specific chemical reagents, that lead to the change in the base‐pairing manner of the metabolically incorporated nucleoside. In this way, nascent RNA information can be directly extracted from the sequencing data of total RNA by tracking and segregating the sequencing fragments, which contain anticipated base mutations.

Thus far, only limited methods have been developed for enrichment‐free nascent RNA detection.^[^ ^10^, ^11^, ^12^, ^13^ ^]^ SLAM seq employs a nucleophilic substitution chemistry to alkylate s^4^U, inducing s^4^U‐to‐C mutation in a reverse transcription (RT)‐dependent manner.^[^ ^10^ ^]^ TimeLapse‐seq transformed s^4^U into cytidine derivative underlying an oxidative nucleophilic aromatic substitution reaction,^[^ ^11^ ^]^ that has also been successfully extended to recode s^6^G to A analogue.^[^ ^12^ ^]^ This oxidation condition caused certain oxidative damage to guanine,^[^ ^11^ ^]^ and TUC‐seq directly converted s^4^U to C by osmium tetroxide and ammonia,^[^ ^13^ ^]^ while not been applied to transcriptome‐wide sequencing. Herein, we employed a novel method based on a nucleophilic addition chemistry to realize s^4^U‐to‐C conversion in NGS‐compatible manner. This strategy is highly efficient and reliable, and can quantitatively analyze nascent RNA at transcriptome scale with little influence on the base‐paring manner of other nucleosides.

By applying our sequencing strategy, we successfully detected the transcriptome‐wide direct gene targets of one potential anticancer drug PDP,^[^ ^14^ ^]^ which functions by binding and stabilizing G‐quadruplex (G4) structures, a non‐canonical nucleic acid secondary structure formed by guanine‐rich sequences. G4 motifs have been identified overrepresented in promoters, particularly in genes driving cancer and associating with cell proliferation,^[^ ^15^, ^16^ ^]^ that offers G4 targeting a therapeutic advantage since the stabilization of these G4s result in inhibition of the expression of these genes.^[^ ^17^, ^18^, ^19^, ^20^ ^]^ To pursue G4s efficiently as therapeutic targets, it is necessary to uncover the direct gene targets of G4 ligands in vivo in order to understand how G4s involved in cancer intervention and therapeutics.

## 2 Results

### 2.1 Acrylonitrile Efficiently Reacts with s^4^U and Induces s^4^U‐to‐C Conversion In Vitro

**Figure 1:**
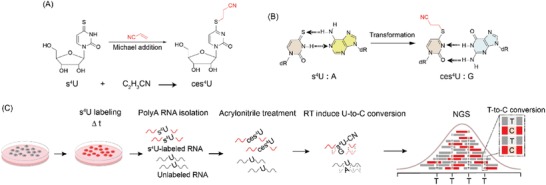
Schematic of AMUC‐seq. A) s^4^U reacted with acrylonitrile to generate ces^4^U through Michael addition. B) s^4^U cyanoethylation changes the hydrogen bonding patterns of s^4^U. After s^4^U cyanoethylation, s^4^U pairs with G instead of A. C) Workflow of AMUC‐seq.

The principle of our method is outlined in **Figure** **1**. Briefly, S‐alkylation of the thiol group of s^4^U in the newly made RNA is caused by the electrophile acrylonitrile to form the S‐cyanoethylated 4‐thiouridine (ces^4^U) via a type of Michael addition under alkaline condition. The cyanoethyl group has been used for thiocarbonyl protection in the synthesis of thiopyrimidine containing oligonucleotide.^[^ ^21^ ^]^ We predicted that the original N3 of s^4^U is no longer a competent proton donor after cyanoethylation, leading to s^4^U inhibiting Watson–Crick base pairing with A. Instead, it forms non‐canonical base pair with G. Taking advantage of this, cyanoethylated s^4^U were replaced by C in cDNA via RT and PCR during sequencing.

Because s^4^U‐to‐C conversions are the identification signals of nascent RNAs, an adequate conversion efficiency is necessary for the recovery of nascent RNAs from total RNAs as complete as it can be. Thus, we first tested the reactivity of the s^4^U cyanoethylation reaction in vitro. We found that the s^4^U‐to‐ces^4^U conversion rate could reach ≈90% under very gentle conditions. By adjusting the reaction conditions, the reaction could be completed within hours (Figure S2A,B, Supporting Information). We compared the selectivity and reactivity of our method with the reported ones (SLAM‐seq^[^ ^10^ ^]^ and TimeLapse‐seq^[^ ^11^ ^]^) all these three methods can achieve high reaction selectivity and efficiency (Figure S3, Supporting Information). Next, we validated the hydrogen bonding patterns of s^4^U and ces^4^U. We prepared s^4^U and ces^4^U‐containing RNA oligos as templates and performed RT‐directed single‐nucleotide incorporation reactions by using RevertAid reverse transcriptase. Undoubtedly, s^4^U preferred to pair with T, yet also has a weak base‐pairing ability toward G, which is probably induced by the tautomeric form of s^4^U. In contrast, only dGTP incorporation was observed opposite to the ces^4^U‐RNA template even after a long reaction time (Figure S2C, Supporting Information). Thus, s^4^U cyanoethylation resulted in a thoroughly s^4^U‐to‐C conversion in the base‐pairing manner.

### 2.2 Acrylonitrile Efficiently Induces s^4^U‐to‐C Conversion in Cells

**Figure 2:**
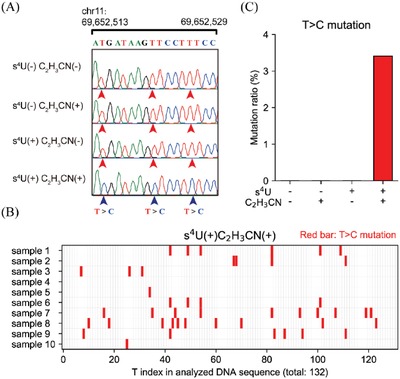
TA‐cloning analysis of s^4^U‐to‐C conversion in untreated *CCND1* mRNA. A) Sanger sequencing results of the specific region (chr 11, 69 652 513–69 652 529). B) The mutation sites of T‐to‐C which indicated the incorporation of s^4^U. C) The average T‐to‐C mutation rate in the selected region (s^4^U(−)C_2_H_3_CN(−), *n* = 16), *CCND1* mRNA treated acrylonitrile (s^4^U(−)C_2_H_3_CN(+), *n* = 24), s^4^U‐tagged *CCND1* mRNA (s^4^U(+)C_2_H_3_CN(−), *n* = 25), and acrylonitrile‐treated s^4^U‐tagged *CCND1* mRNA (s^4^U(+)C_2_H_3_CN(+), *n* = 10). “*n*” stands for the number of TA‐cloning samples used for each statistical analysis.

We next conducted a TA‐cloning study to test the feasibility of our strategy in vivo. We fed HEK293T cells with 50 µm s^4^U for 24 h (a condition before inducing substantial cell death, the cell growth was inhibited by one third due to the prolonged culturing with s^4^U (Figure S4A, Supporting Information).^[^ ^22^, ^23^ ^]^ The isolated total RNAs, treated RNAs with acrylonitrile, amplified specific mRNA (partial of CCND1 mRNA), and then subjected it to TA cloning and Sanger sequencing. Ten clones were selected for mutation analysis. As expected, distinct U‐to‐C transitions appeared only in the acrylonitrile‐treated s^4^U‐RNA populations (**Figure** **2**). Thus, our strategy is feasible for cellular RNA study.

### 2.3 Demonstration of s^4^U‐to‐C Conversion in NGS‐Compatible Manner

**Figure 3:**
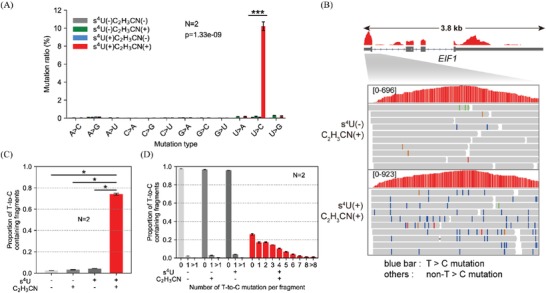
s^4^U labeled RNA detection by acrylonitrile‐mediated s^4^U derivatization and sequencing at transcriptome‐wide scale (AMUC‐seq). A) Mutation rates of all possible mutation types in the sequencing libraries. B) Genome browser view of a representative region displays the base mutations in partial mapping reads of acrylonitrile‐untreated (top) and acrylonitrile‐treated (bottom) s^4^U‐labeled *EIF1* mRNA. C) Quantification of the proportion of T‐to‐C mutation‐containing fragments in each sequencing library. D) Transcriptome‐wide analysis of the distribution of T‐to‐C mutation in each sequencing library.

Inspired by these results, we therefore implemented s^4^U cyanoethylation reaction for s^4^U‐RNA detection on a transcriptome‐wide scale. s^4^U‐labeled mRNAs of HEK293T cells cultured with 50 µm s^4^U for 24 h were prepared for the following tests. First, we performed LC‐MS to measure the reaction efficiency of s^4^U cyanoethylation in total mRNAs. To alleviate the suppression to the s^4^U cyanoethylation reaction imposed by the intricate RNA secondary structures, the purified full‐length mRNAs were fragmented into ≈150 nt RNA fragments prior to acrylonitrile treatment by treating RNA with zinc (II) and heat (70 °C for 10 min), which catalyze the cleavage of RNA by attacking the 2′ hydroxyl group of ribose to produce a mix of 2′ or 3′ phosphate ends with no sequence and structure specificity.^[^ ^24^ ^]^ LC‐MS analysis identified that nearly 90% of s^4^U in total mRNAs converted to ces^4^U (Figure S5, Supporting Information), consisting with the reaction efficiency we tested in vitro on the nucleoside. Then, acrylonitrile‐treated total mRNAs were subjected to library construction and next‐generation sequencing (Figure 1C). The fragment RNAs were reverse transcribed, ligated to adapters, amplified, and deep sequenced. In order to maintain the original strand information of the RNA for accurate gene expression analysis,^[^ ^25^, ^26^ ^]^ we prepared our RNA into directional RNA library, and the time of cDNA synthesis was extended to 50 min in order to reach the complete reverse transcription. Similar to the previous observation, we observed a significant accumulation of T‐to‐C mutations in the mapping reads of the s^4^U‐labeled, acrylonitrile‐treated mRNAs (**Figure** **3**A,B). The frequency of U‐to‐C mutation was calculated to be ≈11 per 100 uridines (Figure 3A). However, the U‐to‐C mutation rates in the control mRNAs (the untreated mRNAs, only s^4^U‐labeled mRNAs, and only acrylonitrile‐treated mRNAs) were kept at low background levels. What is more, all the non‐U‐to‐C mutations in four samples were kept at background levels. These results identified s^4^U or acrylonitrile had little impact on the base‐pairing patterns of all the other nucleotides. In addition, the RNA expression patterns displayed a good correlation between the acrylonitrile‐treated and untreated RNAs (Figure S6B, Supporting Information), suggesting acrylonitrile treatment has an inappreciable influence on the analysis of gene expression.

### 2.4 Retrieving s^4^U Labeled RNA Sequencing Data

We then dissected the s^4^U labeled RNA information from total mRNA sequencing reads by tracking the U‐to‐C transition signals and retracting SNPs, and analyzed the s^4^U labeled RNA recovery efficiency. After 24 h s^4^U labeling, 75% of the mapping fragments have been marked by the desired T‐to‐C mutations (Figure 3C,D), indicating the turnover of the majority of mRNAs. This is reasonable because the median half‐life (*t*_1/2_) of human mRNAs has been suggested to be a few hours.^[^ ^27^ ^]^ We then checked the mapping reads of several fast‐decayed mRNAs in *BCL‐2* family whose *t*_1/2_ have been estimated to be 1–4 h in human B‐cells.^[^ ^27^ ^]^ In theory, the pre‐existing fraction of short‐lived transcripts ought to be completely decayed within the 24 h s^4^U‐incubation time. Indeed, 88.5–95.6% of their mapping fragments contained T‐to‐C conversions (Figures S7 and S8, Supporting Information). The same analysis was also applied to the extremely stable transcripts *APLP2*, *MYH9*, *FLNA*, *MSN*, *CRTAP* and *PRDX5*. These transcripts have been examined to be long‐lived, with *t*_1/2_ in excess of 12 h, in human cells.^[^ ^27^ ^]^ A large fraction of pre‐existing mRNAs was still detected in the total mapping reads (Figures S7 and S8, Supporting Information). These results indicate our method can efficiently recover the s^4^U labeled RNA information from total RNAs.

### 2.5 Measurement of the mRNA Half‐Lives in Human Cells

**Figure 4:**
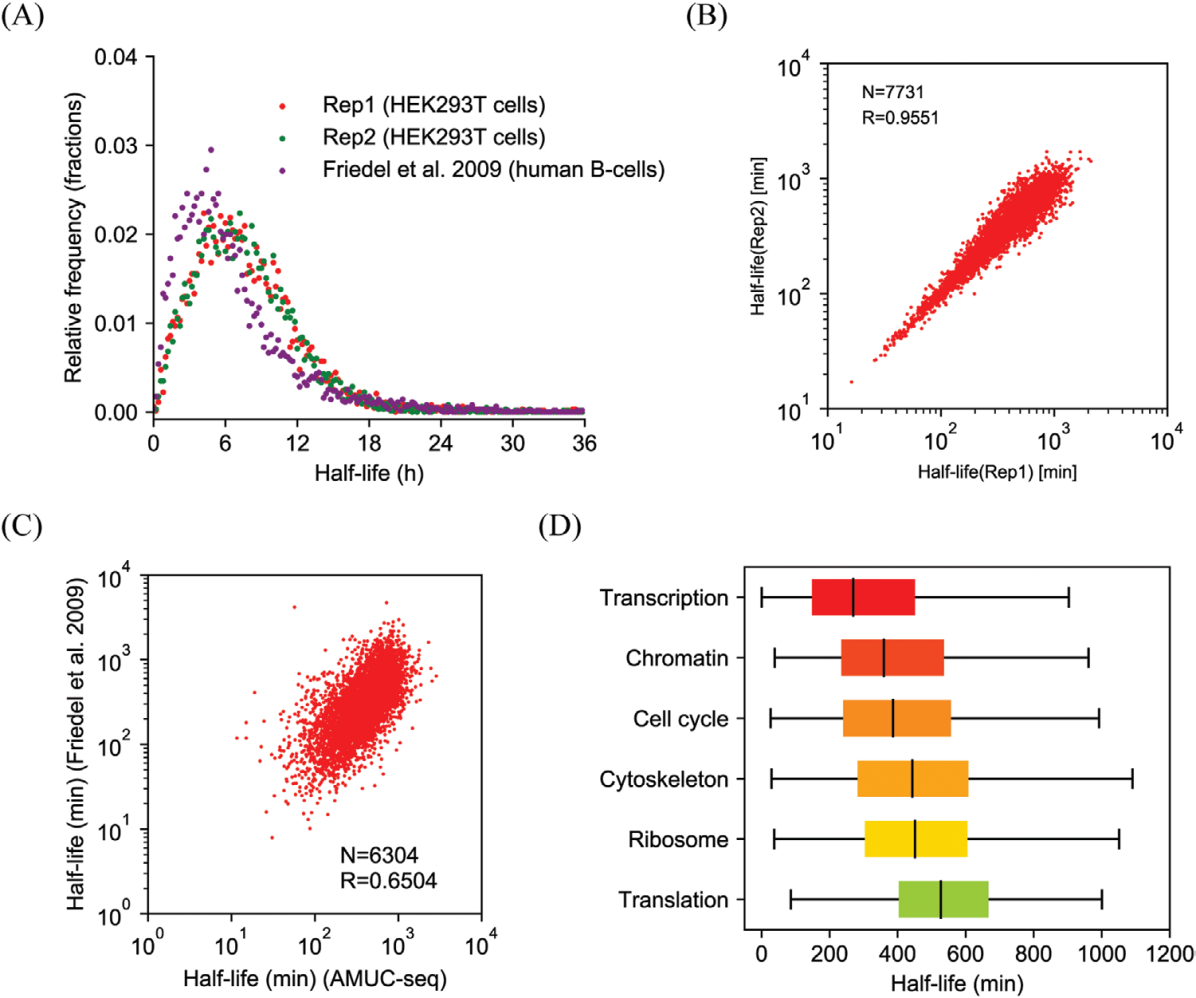
Determination of mRNA half‐lives in HEK293T cells by AMUC‐seq. A) Distribution of RNA half‐lives and the correlation of estimated half‐lives from our method compared to that from Friedel et al.^[^ ^27^ ^]^ B) Correlation of mRNA half‐life measurement between two replicates in HEK293T cells. C) Correlation of mRNA half‐lives estimated by our nascent‐RNA detection strategy compared to that estimated in Friedel et al.^[^ ^27^ ^]^ D) Gene ontology analysis of estimated half‐lives for HEK293T mRNAs. Spearman correlation coefficient (*R*) and transcript counts (*N*) are indicated.

To demonstrate whether our sequencing strategy was robust for studying RNA dynamics, we measured the mRNA stabilities in HEK293T cells through estimating the fraction of newly made RNAs according to the previously reported method.^[^ ^27^ ^]^ RNA stability is determined by the interplay of RNA synthesis, processing, and decay.^[^ ^28^, ^29^ ^]^ Results showed that the median mRNA *t*_1/2_ in HEK293T cells was calculated to be 5.5 h, which correlated with previous observations in human B‐cells (*t*_1/2_ = 5.3 h, **Figure** **4**A–C, Table S1, Supporting Information).^[^ ^27^ ^]^ Consistent with the reported result, the turnover rates of mRNAs responsible for transcription regulation are generally faster than those of mRNAs that are responsible for cell cycle regulation and translation regulation (Figure 4D).^[^ ^27^ ^]^ These results proved our method was adequate for studying cellular RNA dynamics. In addition, because the efficient recovery of nascent mRNA information from total mRNAs is a requirement for the precise measurement of mRNA half‐lives, these results also reidentified AMUC‐seq can high‐efficiently recover nascent RNA information.

### 2.6 Identification of the Direct Gene Targets of a G‐Quadruplex‐Interacting Drug in Human Cells

**Figure 5:**
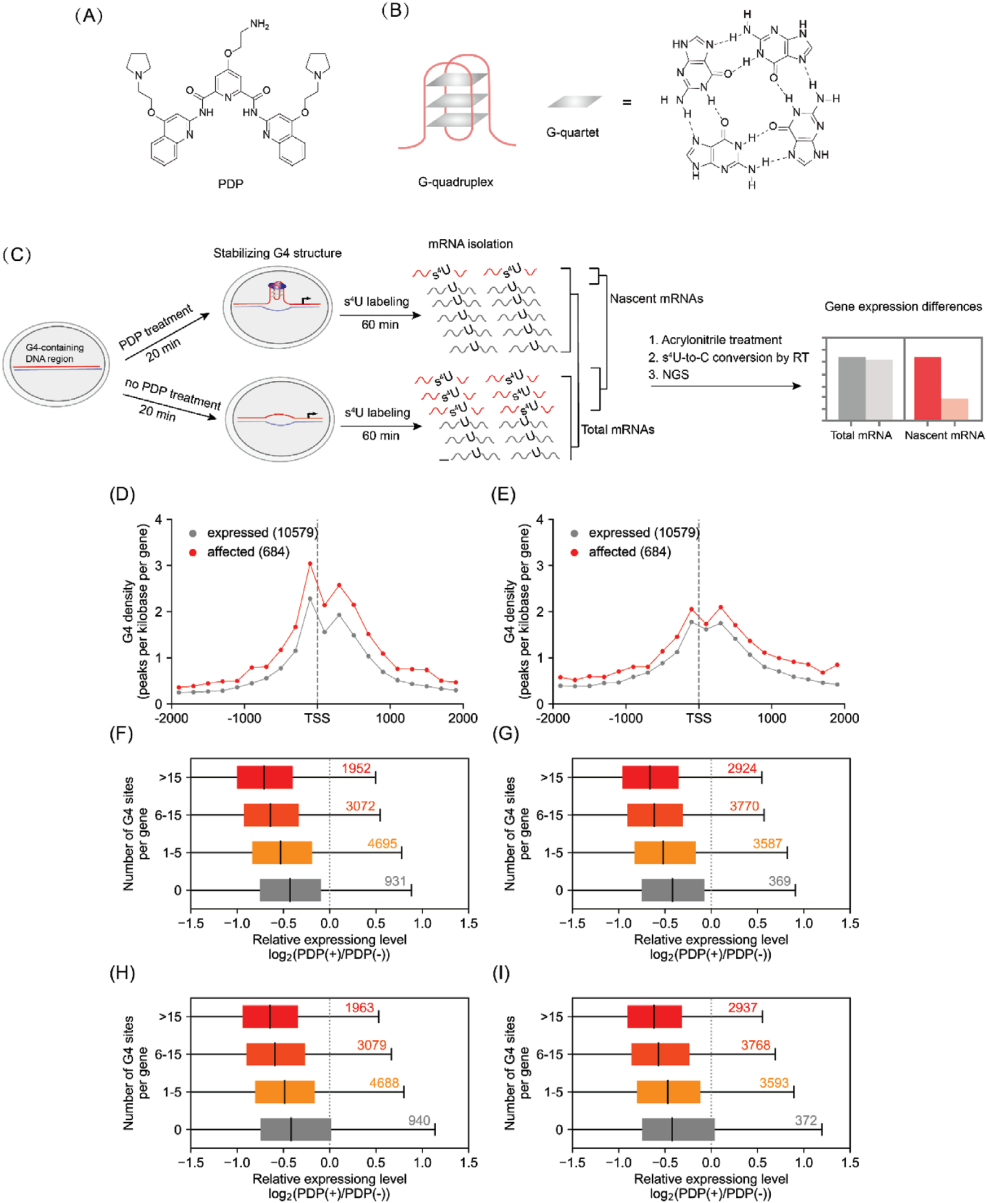
Discovery of the direct gene targets of PDP in HEK293T cells by AMUC‐seq. A) Molecular structure of PDP. B) One antiparallel G‐quadruplex model and the structure of G‐quartet. C) Workflow of our method for analyzing the gene targets of PDP. D,E) Comparison of G4 density at the TSS junction. F–I) Globally comparing the expression level changes after PDP treatment in genes with or without G4 site(s). Data of replication No. 1 (F,G), data of replication No. 2 (H,I). G4 sites were predicted based on G‐quadruplex search algorithm G_3+_N_1–7_G_3+_N_1–7_G_3+_N_1–7_G_3+_ (D,F,H).^[^ ^34^ ^]^ PQS, putative quadruplex sequence. G4 sites were derived from G4‐seq (E,G,I).^[^ ^35^ ^]^ PDP(−)/PDP(+): 0/8 µm PDP treatment.

In order to further exemplify the utility of AMUC‐seq, we tried to apply AMUC‐seq to investigate the direct gene targets of a G‐quadruplex (G4)‐interacting drug PDP (**Figure** **5**A), a pyridostatin analog, in human cells. Discovering drug targets is critical for elucidating the primary mechanism‐of‐action of a drug and predicting its side effects. However, identifying drug targets via gene expression analyses generally requires sufficient drug treatment to achieve detectable cellular responses. At this time, the exact regulatory mechanism can be confused by the derived secondary signaling events. A solution for this is to capture the immediate drug response before the increase in indirect effects. G4 is a non‐canonical nucleic acid secondary structure formed by guanine‐rich sequences that participates in the regulation of diverse biological processes (Figure 5B).^[^ ^30^ ^]^ G4 stabilizers have long been regarded as potential antitumor drugs, mainly due to their inhibition effects to oncogene transcriptions and telomere elongation.^[^ ^31^, ^32^ ^]^ PDP is a small chemical molecule that selectively binds and stabilizes G4 structures.^[^ ^33^ ^]^ In this study, we investigated the direct gene targets of PDP in HEK293T cells by examining the immediate disturbance in mRNA outputs after a short‐term PDP treatment by AMUC‐seq.

The experimental approach is outlined in Figure 5C. At this condition, the drug had no significant effect on the cell cycles and weak toxicity to cells (Figures S9 and S10, Supporting Information). We first tested in HEK293T cells, an immortalized cell line. Because gene transcriptions can usually be inhibited by the stabilization of DNA G4 structures in genes,^[^ ^17^, ^18^, ^19^, ^20^ ^]^ we defined, the genes whose temporal transcription level downregulated were the direct targets of PDP. A short‐term PDP treatment and s^4^U labeling (Figure S4B–D, Supporting Information) revealed 684 downregulated transcripts in the nascent mRNA outputs (Table S2, Supporting Information) (the NGS efficiency is about 80.5% [Figure S11, Supporting Information]), so PDP has a wide range of gene targets in cells. No apparent changes were observed in the total mRNA abundance (Figures S12 and S13, Supporting Information), suggesting nascent RNA detection provides high resolution of RNA dynamics. Gene ontology analysis revealed that those downregulated genes were enriched in genes involved in transcription regulation and kinase activity and development, while lacked in genes involved in olfactory receptor activity, and G‐protein coupled receptor activity (Table S3, Supporting Information).^[^ ^34^ ^]^

Next, we estimated the targeting specificity of PDP by analyzing the G4 contents and G4 distributions in these downregulated genes. These downregulated genes had significantly higher G4 density across the genes, compared to the unaffected gene and upregulated genes (Figure 5D,E; Figure S14, Supporting Information). And the distribution of downregulated genes in genome showed a tendency toward chromosome 16, 17, and 19 (Figure S15, Supporting Information), which have higher G4 frequencies compared with other chromosomes.^[^ ^36^ ^]^ We also have compared the transcription levels of all detected G4‐containing genes to that of detected genes containing no G4s. On the whole, the transcription levels of genes without G4s have no obvious changes, while genes containing G4 sites (from their promoters to transcriptional termination sites) were globally downregulated, the downregulation levels were correlated with the number of G4 sites (Figure 5F–I). These results indicated PDP targeted toward G4s with high selectivity.

To assess the PDP effects in cancer, we focused on the cancer genes in these downregulated genes by searching downregulated genes in a cancer gene database summarized by Bushman.^[^ ^37^ ^]^ We detected a significant enrichment of cancer genes in these downregulated genes, 111 genes were cancer genes (Table S2, Supporting Information), such as *TERT, PIM2*, and *VEGFA*, which have been evidenced to be regulated via G‐quadruplex and involved in the anticancer therapy of G4 stabilizers,^[^ ^36^, ^38^, ^39^ ^]^ and *BAD, AKT2*, and *NOTCH3* which have not yet been studied to be regulated via G4s in these genes, these cancer genes may also be the candidates that involved in the anticancer therapy of PDP. We also have conducted the same experiment in MCF7 cells, a breast cancer cell line, and achieved quite similar results (Figures S15, S18–S20 and Tables S3 and S6, Supporting Information).

### 2.7 G‐Quadruplex‐Interacting Drug Impairs mRNA Maturation

Except for inhibiting gene transcription, we found that PDP can also interfere with the normal 3′‐end polyadenylation process of the transcribed pre‐mRNAs. After PDP treatment, 724 genes (involving 124 cancer genes) displayed distinctly enhanced transcription levels downstream their annotated polyadenylation sites (PASs) (Figure S16 and Table S4, Supporting Information). This result indicated that PDP hindered the pre‐mRNA polyadenylation at their annotated PASs, instead, they skipped these PASs and may be terminated at another PAS behind, that led these aberrant mRNAs had longer 3′ UTRs. The 3′ ends of mRNAs are closely related to mRNA stability, localization, and translation, disruption in the normal polyadenylation processes of mRNAs can lead to mRNA dysfunctions.^[^ ^40^ ^]^ A large fraction of these genes acts in ribosome biogenesis, translation initiation, mRNA splicing, and transcription factor activity (Table S5, Supporting Information), suggesting PDP can disrupt mRNA transcription and protein translation by disturbing the normal polyadenylation processes of these mRNAs.The annotated PASs of these affected mRNAs were remarkably enriched with RNA G4 (Figure S17, Supporting Information), indicating PDP disrupted their normal polyadenylation processes by interacting these RNA G4s. So these mRNAs are also the direct targets of PDP. The same results have also been confirmed in the MCF7 cell line (Figure S21 and Tables S5 and S7, Supporting Information).

## 3 Conclusion

Taken together, we presented a new chemical method for enrichment‐free nascent RNA sequencing. Our experiments demonstrated that acrylonitrile‐mediated s^4^U‐to‐C mutation permits the recognition of metabolically labeled RNA at 1 bp resolution without additional RNA‐purification processes. The high reactivity of this method allowed for efficient recovery of the nascent RNA information. It is noteworthy that acrylonitrile treatment caused negligible non‐s^4^U‐to‐C mutations and undesired U‐to‐C mutations, such as the resulted G‐to‐T mutation rising from the oxidation of guanine to 8‐oxo‐guanine by using TimeLapse‐seq. This enrichment‐free nascent‐RNA analysis method is qualified to transcriptome‐wide study of the temporal RNA kinetics. Except for evaluating the stability of protein‐coding RNAs in human cells, we also have successfully investigated the direct gene targets of a G4‐interactive small molecule. In addition to the well‐known transcriptional regulation effects, we evidenced the blocking of G4 structures also affected the 3′ end polyadenylation of mRNAs that may disrupt gene expression. Both regulatory roles of G‐quadruplex‐interacting drug could be involved in their anticancer therapy. These results could be helpful for the future understanding of the anticancer mechanisms of this kind of potential antitumor drugs.

To the best of our knowledge, this is the first use of nascent RNA sequencing to investigate the active targets of transcription‐modulating drugs. Compared to the conventional analysis methods, enrichment‐free nascent RNA sequencing provides much higher resolution of RNA dynamics that is adequate for defining the primary therapeutic mechanisms of transcription‐modulating drugs, because it can precisely define their direct gene targets and simultaneously identify their transcriptional regulatory effects. We believe our method can be applied to a broader range of applications in biological research, biomedical research, and chemical genomics and has great potential to facilitate the progresses of these fields.

## 4 Experimental Section

##### HPLC Analysis of s^4^U to Cyanoethylated s^4^U

10 µg mL^−1^ s^4^U were reacted in the presence of 306, 764, or 1146 mm acrylonitrile under mild reaction condition (50 mm NaHCO_3_/Na_2_CO_3_, pH 9.5, reaction volume is 100 µL) at indicated temperature for different time periods. The samples were separated on Agilent HPLC system (Thermo Fisher Scientific), employing a Thermo Scientific Hypersil ODS Column (250 mm × 4.6 mm, 5 µm, C18) with a flow rate of 1 mL min^−1^ at 35 °C. eluent A: 0.1 m TEAA (pH 7.0), eluent B: acetonitrile; gradient: 5−40% B in A within 20 min and UV detection at 260 nm.

##### Primer Extension Assay

300 ng RNA oligo (Tempalte‐s^4^U) was treated with acrylonitrile and purified by HPLC. Purified chemically treated RNA (Template‐ces^4^U, 5′‐AGces^4^UCUGCCACAUGCUGCAC‐3′) was then annealed to a FAM 5′ end‐labeled primer, and reverse transcription was performed using RevertAid First Strand cDNA Synthesis Kit (42 °C). The reaction was then stopped and subjected to 20% denaturing PAGE.

##### Cell Viability Assay

12 000 HEK293T cells were seeded per 96 well and grown at 37 °C in DMEM containing 10% FBS and 1% P/S for 24 h. Then, indicated concentrations of s^4^U/PDP were added to the media for indicated time periods. Control cells (treated with only solvents) were prepared under the exactly same conditions. After the incubation period, cell survival was evaluated using MTT assay. 10 µL aliquot of MTT solution (5 mg mL^−1^ in PBS) was added to each well. After 4 h of incubation, the medium was replaced and supplemented with 100 µL of DMSO. The absorbance at 492 nm was measured for each well. Data were expressed as mean values of three individual experiments conducted in duplicate.

##### Cell Cycle Profile and Apoptosis Analysis

100 000 cells were seeded per 24 well and grown at 37 °C in DMEM containing 10% FBS and 1% P/S for 24 h. Then, indicated concentrations of s^4^U/PDP were added to the media for indicated time periods. Control cells (treated with only solvents) were prepared under the exactly same conditions. After the incubation period, the culture media was removed, rinsed the cells with PBS and added trypsin to dissociate cells. The culture media and PBS rinse were pooled and saved. Once cells were fully trypsinized, the media was quenched with the saved media and PBS wash from the previous step. Cell cycle profile and apoptosis analysis were then performed.

##### Cell Cycle Profile

Cells were fixed in 66% ethanol and stored at +4 °C for 2 h. 50 000–100 000 resuspended cells were taken, centrifuged at 1000 g for 5 min, discarded the supernatant, and rehydrated cells in PBS. Cells were the stained with propidium iodide + RNase for 30 min (Immediately prior to use prepare the Propidium Iodide + RNase staining solution in PBS). For flow cytometry detection, propidium iodide fluorescence intensity on FL2 (660 nm) of a flow cytometer and 488 nm laser excitation was collected.

Apoptosis analysis: 50 000–100 000 resuspended cells were taken, centrifuged at 1000 g for 5 min, discarded the supernatant, and added 195 µL of Annexin V‐FITC binding solution to gently resuspend the cells. 5 µL Annexin V‐FITC was added and mixed gently then 10 µL of propidium iodide staining solution was added and mixed gently. Incubated at room temperature in the dark for 10–20 min, then placed in an ice bath. For flow cytometry detection Annexin FITC fluorescence intensity on FL1 (530 nm) and propidium iodide fluorescence intensity on FL2 (660 nm) of a flow cytometer and 488 nm laser excitation were collected.

##### TA Cloning Analysis

3 × 10^6^ HEK293T cells were seeded per 10 cm cell dish and grown for 24 h. Then, s^4^U was added to the medium at a final concertation of 50 µm and grown for another 24 h. Total RNA was extracted using TRIzol reagent (Invitrogen) according to the manufacture's protocol and the RNA concentration was determined by Nanodrop (Thermo Fisher). gDNA was removed by TURBO DNase (Invitrogen), then RNA was purified by RNA Clean & Concentrator Kits (Zymo Research). Isolated RNA was then subjected to s^4^U cyanoethylation reaction. After reacted with acrylonitrile, RNA was purified by RNA Clean & Concentrator Kits. 500 ng purified RNA was reverse transcribed using PrimeScript RT Master Mix (TaKaRa). The resulting cDNA was then amplified with Taq DNA polymerase (Thermo Fisher) using *CCND1*‐specific primers (forward primer: GAGGGCAGTTTTCTAATGGA; reverse primer: GAGGGCAGTTTTCTAATGGA). PCR products were purified by gel extraction. Isolated PCR products were further applied to TA cloning analysis by using pEASY‐T1 Simple Cloning Kit (TransGen Biotech). For each sample, 30 clones were selected for Sanger sequencing.

##### HPLC‐MS Analysis

3 × 10^6^ HEK293T cells were seeded per 10 cm cell dish and grown for 24 h. Total RNA was extracted after that 50 µm s^4^U was added to the medium and grown for another 24 h. Polyadenylated RNA was isolated by subjecting total RNA to oligo(dT) enrichment using Dynabeads Oligo(dT)_25_ (NEB) following manufacturer's instructions and then gDNA was removed. Purified polyadenylated RNA was fragmented using RNA fragmentation reagents (Invitrogen) at 70 °C for 10 min, then stopped by stop solution and purified by RNA Clean & Concentrator Kits. 2 µg RNA fragments were subjected to s^4^U cyanoethylation reaction at 45, 50, and 55 °C for different time periods. To enzymatically degrade RNA to monomeric ribonucleosides, Nuclease P1 (2 U, Sigma) was added to a 50 µL solution containing 2 mm ZnCl_2_, 10 mm NaCl, 100 µm DTT, and 600 ng purified RNA and incubated at 37 °C for 2 h. Then, 6 µL 200 mm Tris‐HCl (pH 7.9), 1 µL 1 mm DTT, 3 µL 100 mm MgCl_2_, and 2 µL Shrimp Alkaline Phosphatase (2 U, NEB) were added to the reaction solution and incubated for another 2 h at 37 °C. Each sample was centrifuged at 12 000 g at 4 °C for 20 min, 50 µL supernatant was collected and directly subjected to HPLC‐MS analysis. s^4^U and ces^4^U ribonucleosides which diluted in enzymatic buffer containing a mixture of four common bases were used to make a standard curve for quantitative MS analysis. Standards were prepared as Table S2, Supporting Information.

The samples were separated on a Shimadzu HPLC system (LCMS‐8050), employing a ZORBAX Rapid Resolution High Definition column (50 mm × 2.1 mm; 1.8 µm; C18) with a flow rate of 200 µL min^−1^. Nucleosides were on‐line analyzed using a triple quadrupole mass spectrometer after electrospray ionization with the following multiple reaction monitoring: s^4^U *m*/*z* 260→129, and ces^4^U *m*/*z* 318→129.

##### RNA Library Preparation

3 × 10^6^ HEK293T cells were seeded per 10 cm cell dish and grown for 24 h. For T > C conversion identification, 50 µm s^4^U was added to the medium and grown for another 24 h. For PDP‐mediated RNA dynamic analysis, PDP was added to the medium for 20 min prior to 1 h 500 µm s^4^U incubation. After that, RNA samples for library construction were prepared by total RNA extraction, polyadenylated RNA isolation, gDNA removal, RNA fragmentation, and RNA cyanoethylation (45 °C, 10 h). Standard RNA seq libraries were prepared using NEBNext Ultra II Directional RNA Library Prep Kit for Illumina (NEB) according to manufacturer's instructions. Sequencing was performed on Illumina HiSeq X Ten.

##### Sequencing Alignment

All library samples shared the same processing methods in the initial few steps. First, all the paired‐end reads from NGS technology were trimmed using cutadapt to remove Illumina adapter sequences with parameters “‐m 20 –max‐n 3 ‐q 30”.^[^ ^41^ ^]^ Then, only one read from reads group with identical sequence is kept to remove potential PCR amplification bias.^[^ ^42^ ^]^ Next, the remaining reads were mapped to human ribosome RNA sequences (5.8S, 46S,12S, and 16S) using bowtie2.^[^ ^43^ ^]^ The paired‐end reads that failed to map to rRNA were finally mapped to human genome (GRCh37) using hisat2 with parameter “–mp 2,2 –rna‐strandness FR –no‐mixed –no‐discordant –seed 1234” (index of hisat2 was download from hisat2 office website).^[^ ^44^ ^]^ Only the proper pair and uniquely mapped alignments was persisted for the downstream pipelines.

##### Mismatch Site Determination

Mismatch events were extracted from alignment results by custom script. For two mismatch events with the same position and mismatch type extracted from read 1 and read 2 in the same fragment, only the one with the best sequencing quality was retained. Mismatch events induced by SNP or with lower sequencing quality than 41 were excluded. For specific mismatch site, if the count and ratio of correspond mismatch event was larger than or equal to 5 and 5% in control samples, respectively, it was treated as a new SNP, and the mismatch events induced by new SNPs were finally excluded from all samples. Counting the number of mismatch events for all mismatch type and the number of reference base, the mismatch ratio was calculated as following formula

(1) $${\text{Ratio}}_{\text{ref/alt}}\;=\;\frac{N_{\text{ref/alt}}}{N_{\text{ref}}}\;$$

where the Ratio_ref/alt_ represents the mismatch ratio of “ref‐to‐alt” (e.g., Ratio_T/C_ represents the mismatch ratio of “T‐to‐C” mutation), *N*_ref/alt_ represents the number of “ref‐to‐alt” mismatch event, and *N*_ref_ represents the expected sequencing number of base “ref”.

##### G‐Quadruplex Distribution Analysis

The potential G4 intervals were extracted from the genome sequences by using custom script. The sequences in these intervals conform to the regular expression “G_3+_N_1‐7_ G_3+_N_1‐7_G_3+_N_1‐7_G_3+_”.^[^ ^34^ ^]^ G4 intervals from G4‐seq were also used to perform analysis.^[^ ^35^ ^]^ The distribution of G4 around TSSs and PASs of each transcripts were calculated.

##### Half‐Life Calculation

For a transcript, a shorter half‐life corresponded to a higher proportion of labeled‐RNA over total‐RNA after s^4^U and C_2_H_3_CN treatment. According to this, the total number of fragments and the number of labeled‐fragments (with T‐C mismatch event) of each transcript were calculated respectively. According to previous reports, use the following formula to calculate the half‐life of the transcript^[^ ^27^ ^]^

(2) $$N_{\text{e}}\;=\;N_{\text{t}}\;-\;N_{\text{n}}$$

(3) $$t_{1/2}\;=\;\frac{-T\;\times \;\text{ln}2}{\ln\left(1-\frac{1}{1\;+\;N_{\text{e}}/N_{\text{n}}}\right)}$$

where *N*_e_ represents the fragment number of unlabeled‐RNA, *N*_t_ represents the fragment number of total‐RNA, *N*_n_ represents the fragment number of labeled‐RNA, and *T* represents the treatment time.

##### Gene Expression Analysis

The expression level of each transcripts was represented by fragment count per kilobase per million fragment (FPKM), which were calculated using below formula

(4) $$\text{FPKM}\;=\;\frac{N\;\times \;10^{9}}{L\;\times \;S}$$

where *N* represents the fragment count, *L* represents the transcript length, and *S* represents the count of all fragment. The calculation of labeled‐FPKM is similar to the calculation of FPKM, except that *C* represents the number of labeled fragments.

##### Identifying PDP Target Genes

In order to identify the target genes of PDP, the fold‐change of FPKM and labeled‐FPKM were calculated for PDP treatment sample versus control sample respectively. The canonical protein coding transcripts that met following criteria at all replicates and comparison group were treated as PDP target genes: a) FPKM of PDP treatment and control ≥1; b) labeled‐FPKM of control ≥1; c) log_2_(labeled‐FPKM of PDP treatment/labeled‐FPKM of control) ≤ ‐log_2_(1.5).

##### Analyzing Impaired mRNA Maturation

The impaired mRNA maturation led to transcriptional read‐through, which resulted in increment of the fragment count of downstream of PAS. Downstream‐FPKM (FPKM of PAS downstream region, +4000 bp) was used to measure the fragment count of PAS downstream and eliminate deviation in library size. Transcripts whose downstream regions overlap in the same direction as other transcripts are not considered. The canonical protein coding transcripts that met following criterions at all replications and comparison group were treated as impairs mRNA maturation: a) FPKM of PDP treatment and control ≥1; b) downstream‐FPKM of PDP treatment ≥0.2; c) ‐log_2_(1.2) ≤ log_2_(FPKM of PDP treatment/FPKM of control) ≤ log_2_(1.2); d) log_2_(downstream‐FPKM of PDP treatment/downstream‐FPKM of control) ≥ log_2_(1.5).

##### Gene Ontology Analysis

GO‐term enrichment analysis was performed using the PANTHER database.^[^ ^45^ ^]^

## Conflict of Interest

The authors declare no conflict of interest.

## Supporting information

Supporting Information
