# Supplementary material for: Acrylonitrile‐Mediated Nascent RNA Sequencing for Transcriptome‐Wide Profiling of Cellular RNA Dynamics
Source: Adv Sci (Weinh). 2020 Mar 5;7(8):1900997. doi: 10.1002/advs.201900997 (PMC7175251; doi:10.1002/advs.201900997)
Supplement: Supplementary file 1 — Supporting Information [file ADVS-7-1900997-s001.pdf]

Copyright WILEY-VCH Verlag GmbH & Co. KGaA, 69469 Weinheim, Germany, 2020.

ADVANCED SCIENCE
Open Access

Supporting Information

for *Adv. Sci.,* DOI: 10.1002/advs.201900997

Acrylonitrile-Mediated Nascent RNA Sequencing for Transcriptome-Wide Profiling of Cellular RNA Dynamics

*Yuqi Chen, Fan Wu, Zonggui Chen, Zhiyong He, Qi Wei, Weiwu Zeng, Kun Chen, Feng Xiao, Yushu Yuan, Xiaocheng Weng, Yu Zhou, and Xiang Zhou**

((Supporting Information can be included here using this template))

Copyright WILEY-VCH Verlag GmbH & Co. KGaA, 69469 Weinheim, Germany, 2018.

Supporting Information

Acrylonitrile-mediated nascent RNA sequencing for transcriptome-wide profiling of cellular RNA dynamics ((no stars))

*Yuqi Chen[1] †, Fan Wu[1] †, Zonggui Chen[3] †, Zhiyong He[1] †, Qi Wei[1], Weiwu Zeng[1], Kun Chen[1], Feng Xiao[1], Yushu Yuan[1], Xiaocheng Weng[1], Yu Zhou[2,3], Xiang Zhou[1,3]**

Synthesis of ces^{4}U: Acrylonitrile was added to a solution of s^{4}U (50 mg, 0.19 mmol) in carbonate/bicarbonate buffer (50 mM $NaHCO_3$/Na_2CO_3, pH 9.5) (1 mL). The reaction solution was stirred at 50℃ for 10 hours. TLC (DCM:MeOH = 10:1) indicated the near-complete consumption of s^{4}U. After removal of solvent by evaporation, the crude product was purified by silica gel chromatography eluted with DCM:MeOH = 10:1 to give product (56 mg, 93%) as a white solid.

^{1}H NMR (400 MHz, DMSO-d_6) δ 8.31 (dd, J = 7.1, 1.9 Hz, 1H), 6.53 (d, J = 7.1 Hz, 1H), 5.74 (d, J = 2.7 Hz, 1H), 5.55 (d, J = 4.8 Hz, 1H), 5.22 (t, J = 5.0 Hz, 1H), 5.07 (d, J = 5.5 Hz, 1H), 4.01 – 3.93 (m, 2H), 3.91 (dt, J = 5.9, 2.7 Hz, 1H), 3.74 (ddd, J = 12.4, 5.3, 2.6 Hz, 1H), 3.59 (ddd, J = 12.4, 5.4, 2.7 Hz, 1H), 3.35 (d, J = 1.5 Hz, 2H), 2.95 (t, J = 6.7 Hz, 2H).

^{13}C NMR (101 MHz, DMSO-d_6) δ 175.24, 153.27, 142.46, 119.71, 103.22, 90.68, 84.60, 74.98, 68.95, 60.11, 60.01, 24.90, 17.90.

MS (HR-ESI) calculated for $C_{12}H_{15}N_3O_5S$ m/z $[M+H]^+$ cal.: 314.08052 found: 314.08130

RNA solid-phase synthesis: Synthesis of s^4U phosphoramidite and s^4U-containin RNA oligo (Template-s^4U, 5′ -AGs^4UUCUGCCACAUGCUGCAC-3′) were performed as previously described (1). To purify synthesized RNA oligo, Crude RNA products were dissolved in DEPC water. The samples were separated on Agilent HPLC system (Thermo Fisher Scientific), employing a Thermo Scientific Hypersil ODS (C18) Column (250 mm × 4.6 mm, 5 µm) with a flow rate of 1 mL/min. eluent A: 0.1 M TEAA or TEAB (pH 7.0), eluent B: acetonitrile; gradient: 5−30% B in A within 30 min and UV detection at 260 nm. Target RNA were evaporated to dryness. The molecular weight was confirmed by LC−ESI mass spectrometry. Nanodrop quantitative analysis also can observe UV absorption at 330 nm (the absorbance of s^4U).

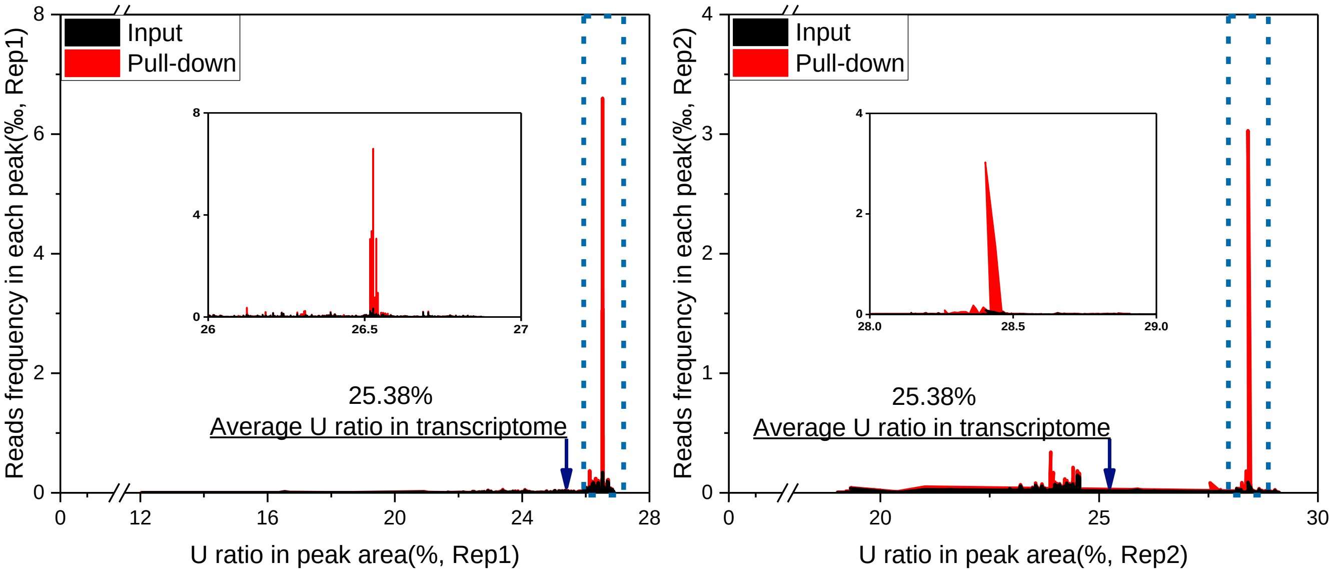

Figure S1. U ratio in peak area of both input and pull-down datasets (two replications), enriched fragments have significantly higher U ratio than non-enriched fragments.

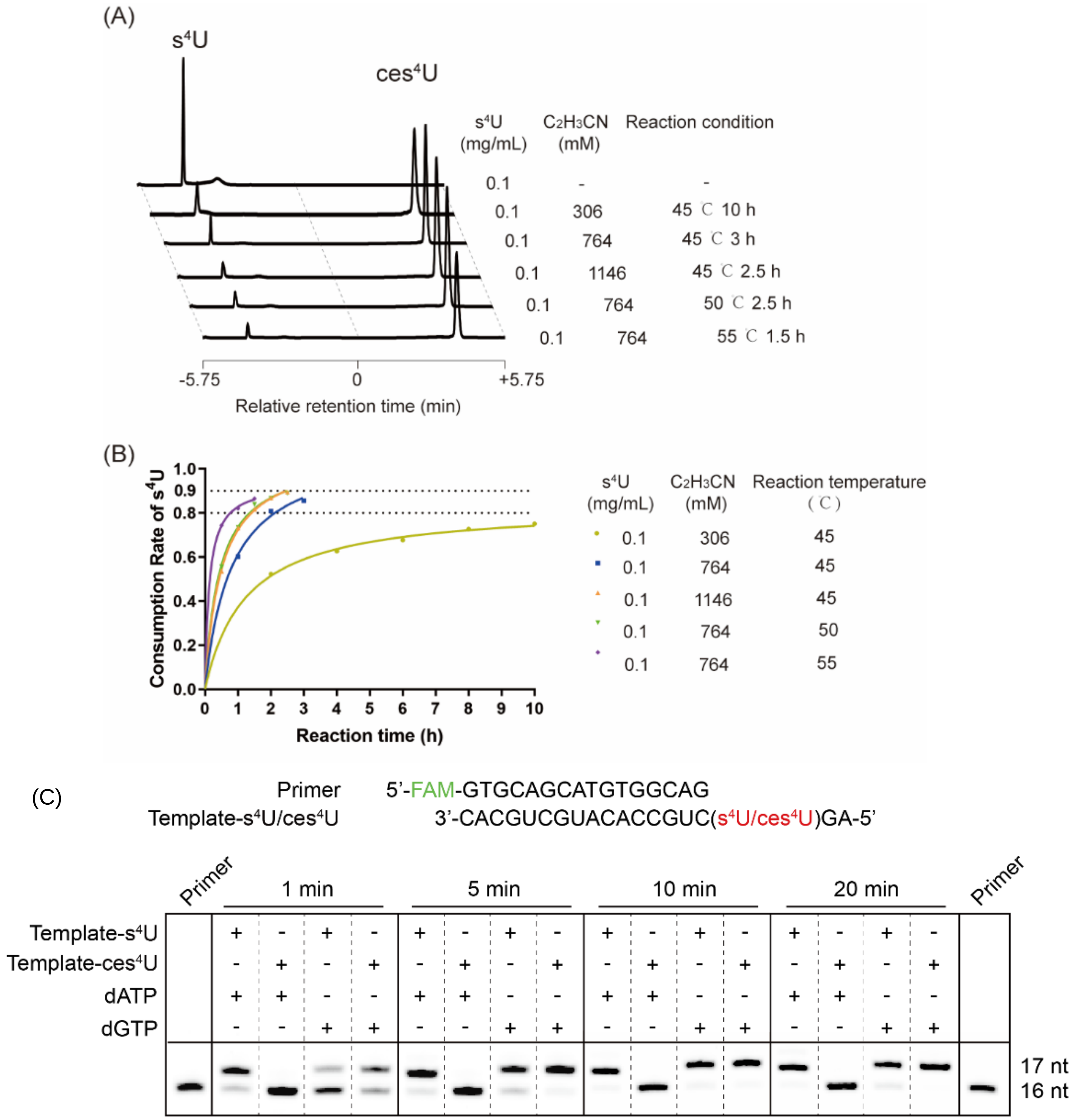

Figure S2. HPLC analysis of the reaction rate of s^{4}U cyanoethylation (A); HPLC detection of the s^{4}U-to-ces^{4}U conversion under various reaction conditions (B); Quantification of the consumption rate of

s^4U under various reaction conditions) and primer extension assay probing the s^4U-to-C conversion in vitro (C).

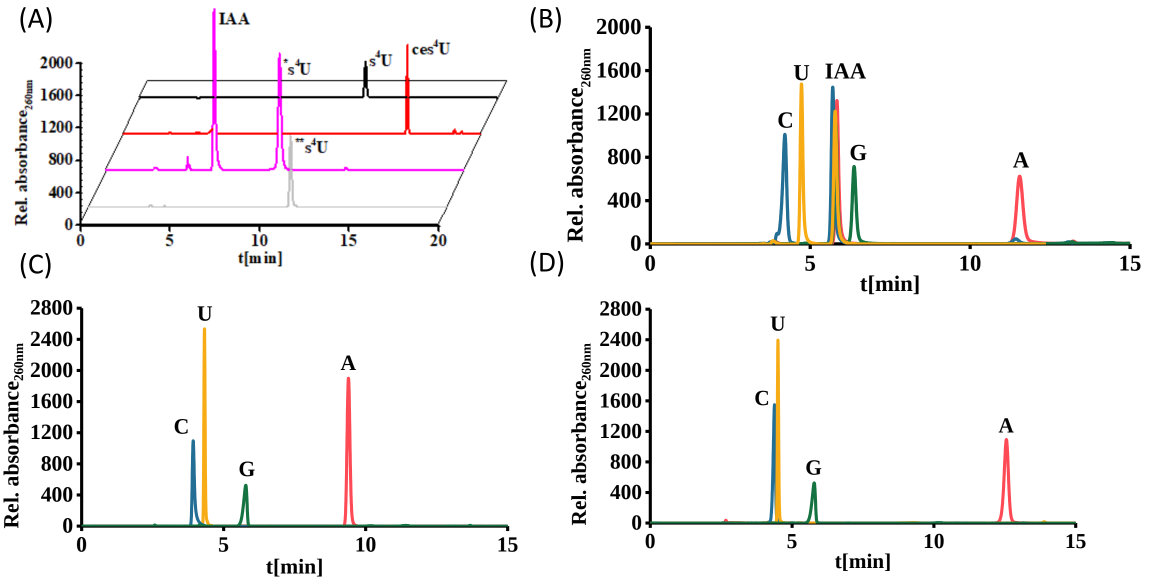

Figure S3. HPLC results of reaction efficiency. (A) ces^{4}U: 4-thiouracil was incubated with acrylonitrile in carbonate-bicarbonate buffer. *s^{4}U: 4-thiouracil was incubated with iodoacetamide. **s^{4}U: 4-thiouracil was incubated with tris(pentafluoroethyl)-amine and oxidant (mCPBA); (B) The nucleotides was incubated with tris(pentafluoroethyl)amine and oxidant (mCPBA)[(2)]; (C) The nucleotides was incubated with iodoacetamide[(3)]; (D) The nucleotides was incubated with acrylonitrile in carbonate-bicarbonate buffer.

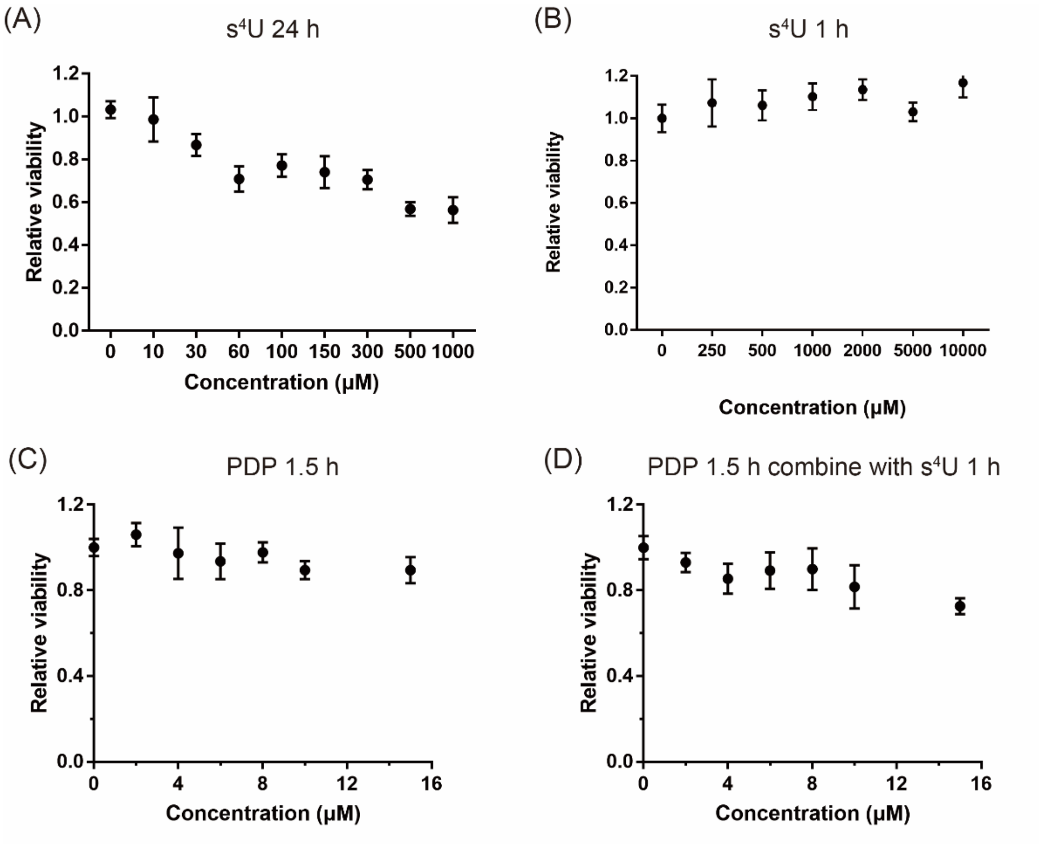

Figure S4. Cell viability assay performed for indicated time periods in the presence of s^4U or PDP or s^4U combining with PDP. (A) Viability of HEK293T cells cultured with increasing concentrations of s^4U (0, 10, 30, 60, 100, 150, 300, 500 and 1000 μM) for 24 h; (B) Viability of HEK293T cells cultured with increasing concentrations of s^4U (0, 250, 500, 1000, 2000, 5000 and 10000 μM) for 1 h; (C) Viability of HEK293T cells cultured with increasing concentrations of PDP (0, 2, 4, 6, 8, 10 and 15 μM) for 1.5 h; (D) Viability of HEK293T cells cultured with increasing concentrations of PDP (0, 2, 4, 6, 8, 10 and 15 μM) for 1.5 h. After cells incubated with PDP for 0.5 h, 500 μM s^4U was added to each well and incubated for another 1 h. Each condition was tested with three individual cell cultures. Error bars indicate mean ± SD of two independent experiments.

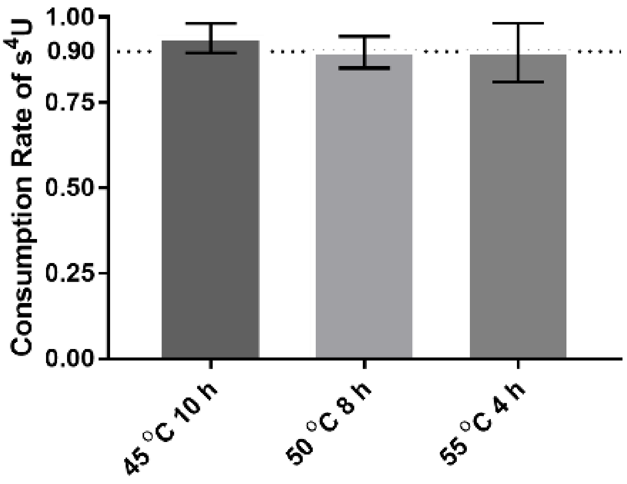

Figure S5. Quantification of s^{4}U-to- ces^{4}U conversion in total polyadenylated RNA of HEK293T cells after 24 h metabolic labeling with 50 µM s^{4}U. s^{4}U-to- ces^{4}U conversion was determined by HPLC-MS analysis following digestion of polyadenylated RNA into monomeric ribonucleosides. Three different reaction conditions were tested (45℃ 10 h, 50℃ 8 h, 55℃ 4 h). Error bars indicate mean ± SD of two independent experiments.

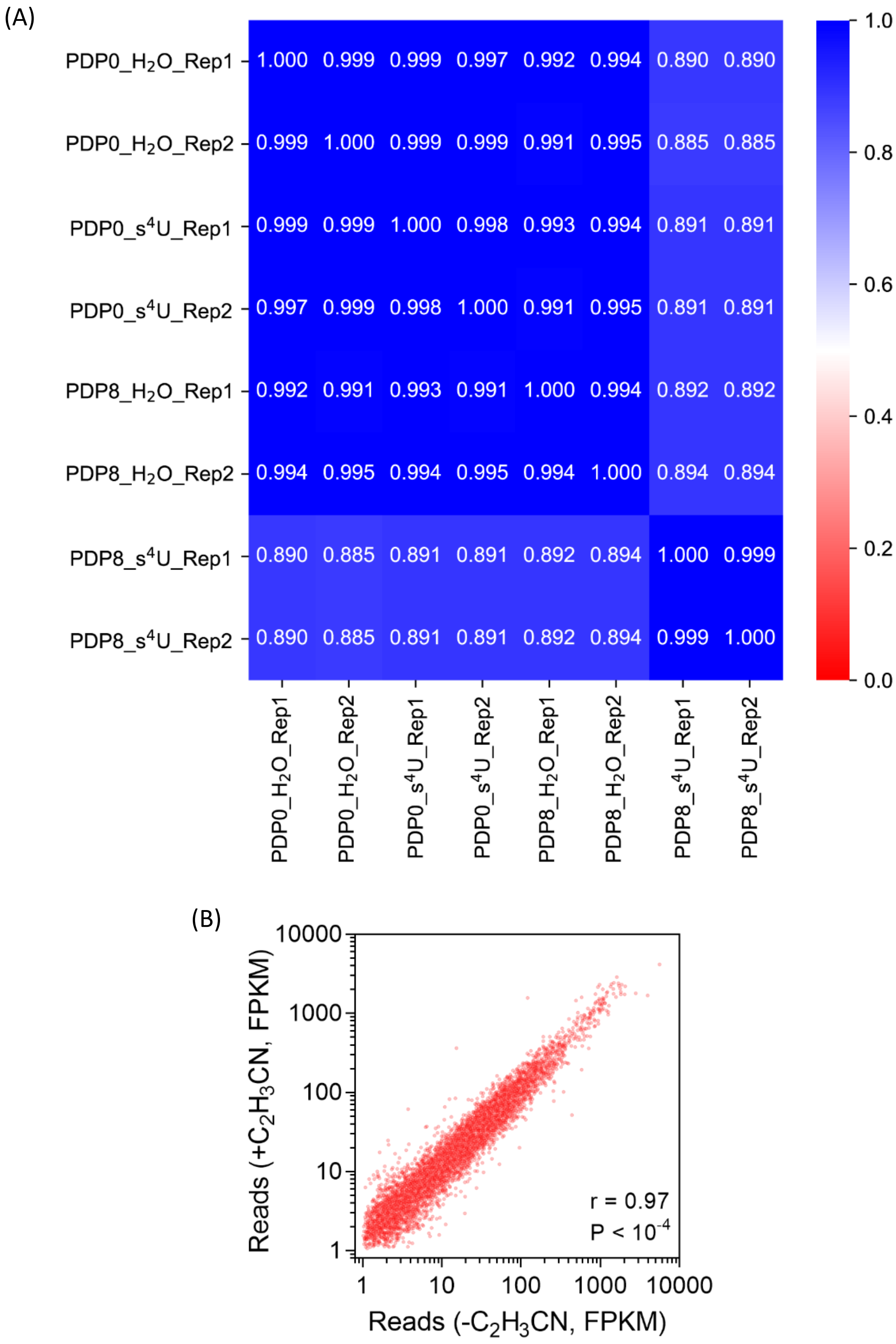

Figure S6. Correlation analysis of estimated RNA expression values. (A) Heatmap of gene expression in MCF-7 of all samples (whether s^4U/PDP is added). (B) Scatter plot of gene expression in HEK293T of all samples (whether it is treated with acrylonitrile or not), spearman correlation coefficient (r), P value and transcript counts are indicated.

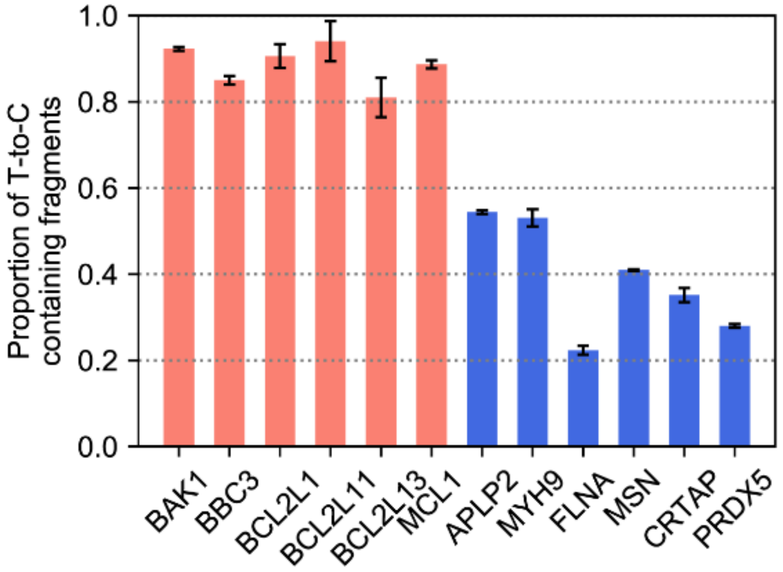

Figure S7. Quantification of the proportion of sequenced reads containing T-to-C mutation mapping to *BAK1*, *BBC3*, *BCL2L1*, *BCL2L11*, *BCL2L13*, *MCL1*, *APLP2*, *MYH9*, *FLNA*, *MSN*, *CRTAP* and *PRDX5*, in each sequencing library. Error bars indicate mean ± SD of two independent experiments. The genes with red bar are those with short half-lives (shorter than 12 h), while the genes with blue bar are those with long half-lives (longer than 12 h).

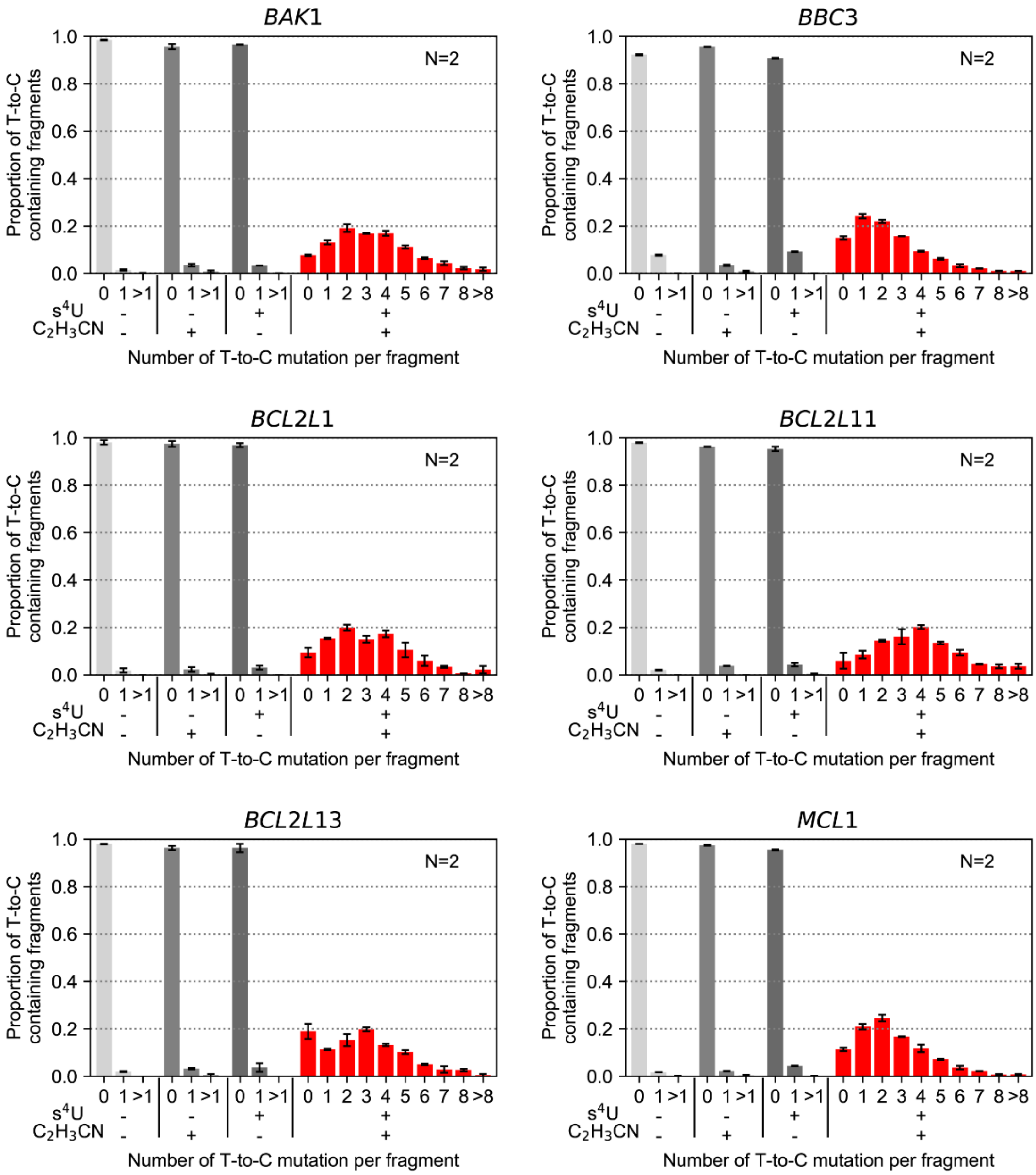
BAK1
BBC3
BCL2L1
BCL2L11
BCL2L13
MCL1
N=2
Proportion of T-to-C containing fragments
1.0
0.8
0.6
0.4
0.2
0.0
0 1 >1 0 1 >1 0 1 >1 0 1 2 3 4 5 6 7 8 >8
s^4U - - + +
C_2H_3CN - + - +
Number of T-to-C mutation per fragment

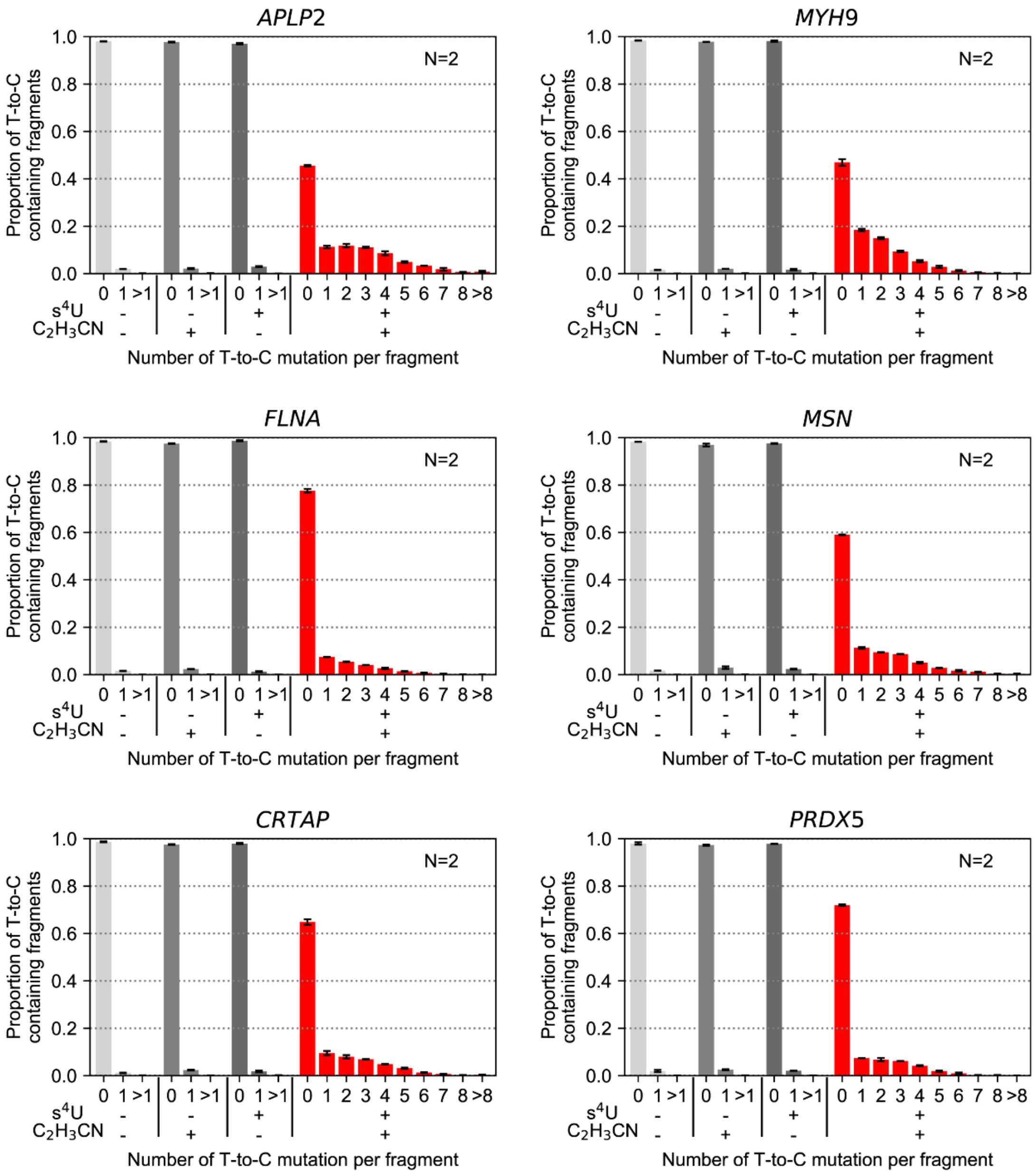

Figure S8. Transcriptome-wide analysis of the distribution of T-to-C mutation in sequenced reads mapping to *BAK1*, *BBC3*, *BCL2L1*, *BCL2L11*, *BCL2L13*, *MCL1*, *APLP2*, *MYH9*, *FLNA*, *MSN*, *CRTAP* and *PRDX5*, in each library prepared from a control polyadenylated RNA sample ($s^4U(-)$ $C_2H_3CN(-)$), a control polyadenylated RNA sample treated acrylonitrile ($s^4U(-)$ $C_2H_3CN(+)$), a s^4U-tagged polyadenylated RNA sample ($s^4U(+)$ $C_2H_3CN(-)$), and acrylonitrile-treated s^4U-tagged polyadenylated RNA sample ($s^4U(+)$ $C_2H_3CN(+)$). Error bars indicate mean ± SD of two independent measurements (Sequencing).

(A1)

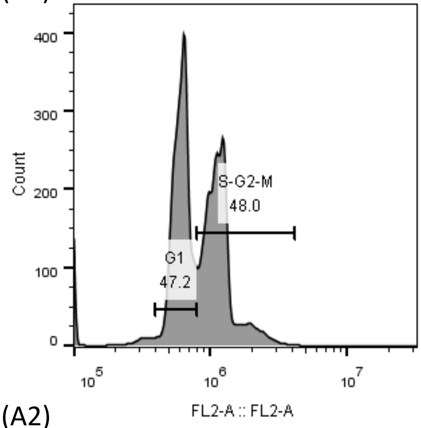

(A2)

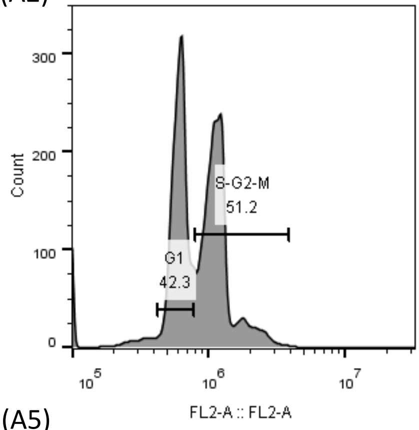

(A3)

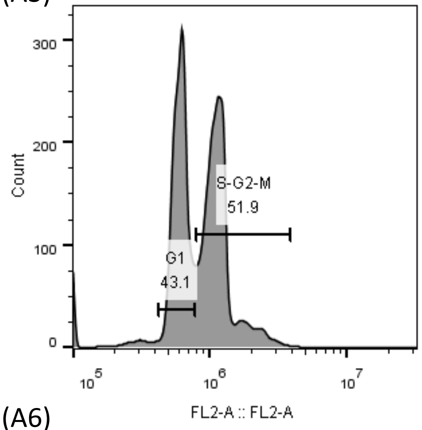

(A4)

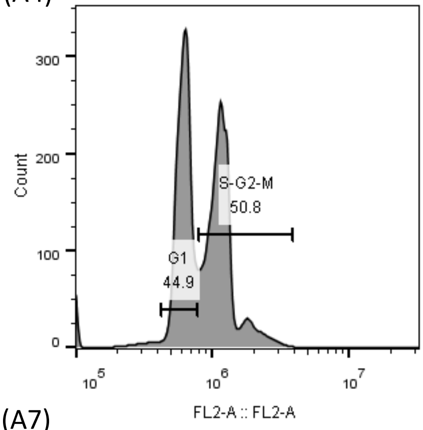

(A5)

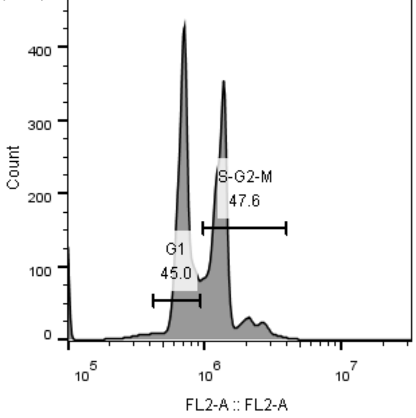

(A6)

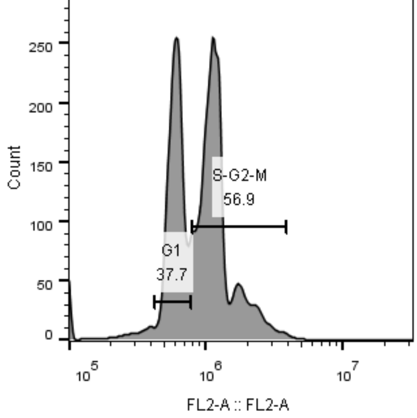

(A7)

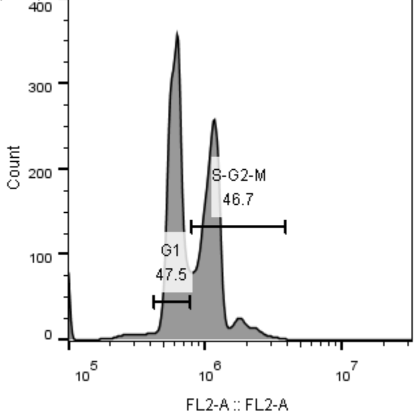

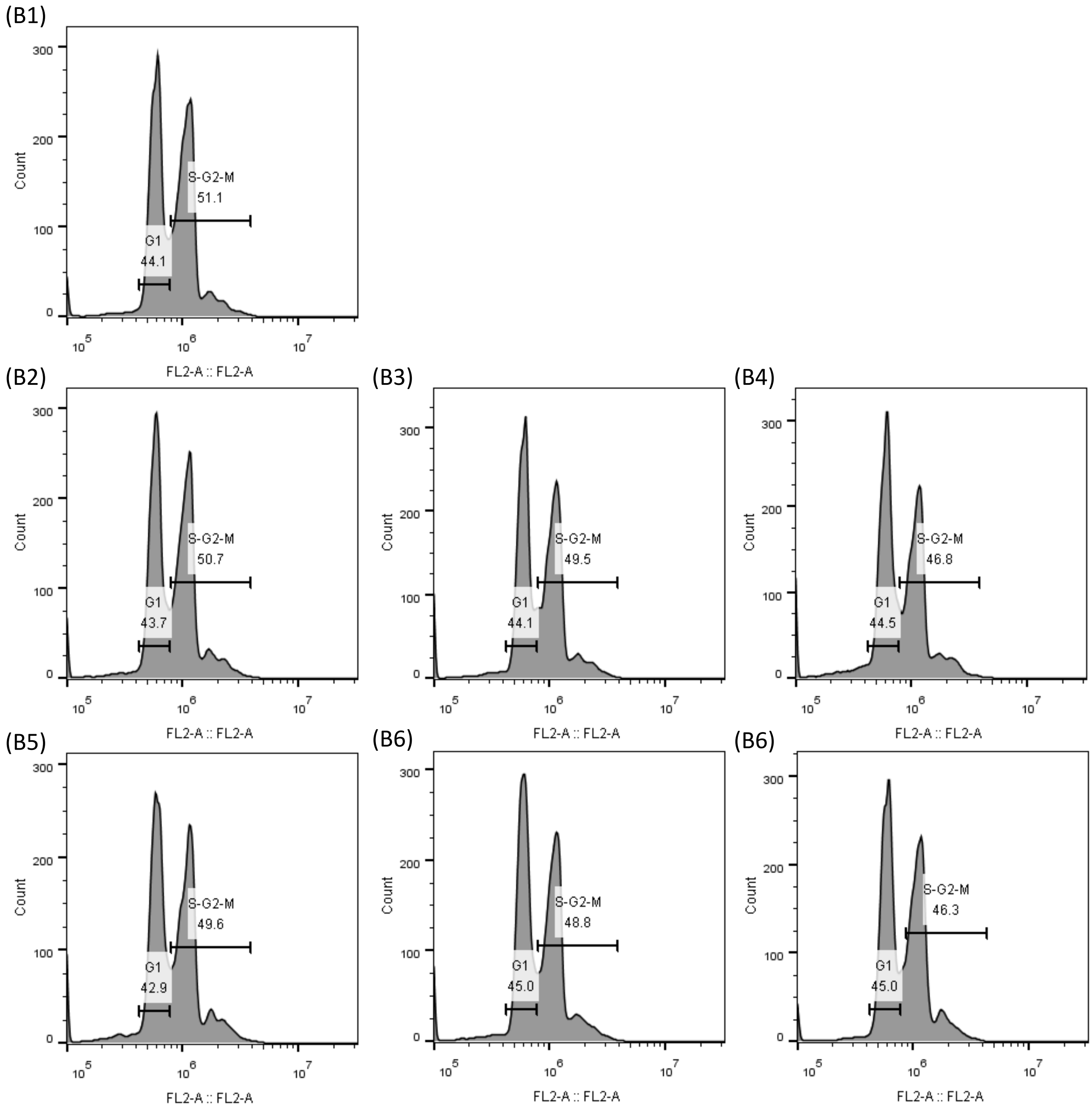
(B1)
Count
S-G2-M
51.1
G1
44.1
FL2-A :: FL2-A
(B2)
S-G2-M
50.7
G1
43.7
(B3)
S-G2-M
49.5
G1
44.1
(B4)
S-G2-M
46.8
G1
44.5
(B5)
S-G2-M
49.6
G1
42.9
(B6)
S-G2-M
48.8
G1
45.0
(B6)
S-G2-M
46.3
G1
45.0

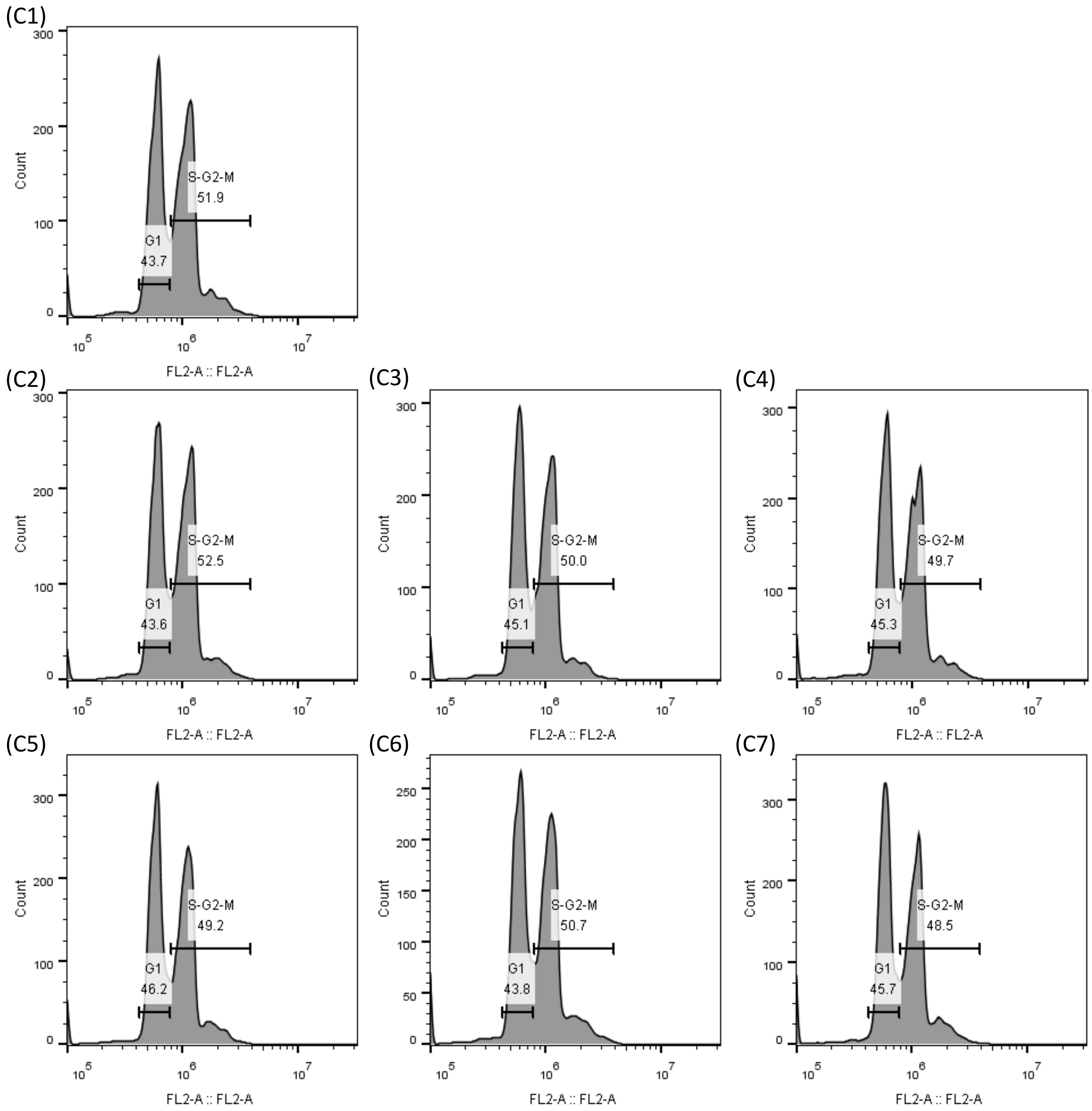

Figure S9. Cell cycle profile in different incubation conditions, (A) cultured with increasing concentrations of s^4U (1-7 stand for concentration of 0, 250, 500, 1000, 2000, 5000 and 10000 μM)

for 1 h; (B) cultured with increasing concentrations of PDP (1-7 stand for concentration of 0, 2, 4, 6, 8, 10 and 15 μM) for 1.5 h; (C) cultured with increasing concentrations of PDP (1-7 stand for concentration of 0, 2, 4, 6, 8, 10 and 15 μM) for 1.5 h. After cells incubated with PDP for 0.5 h, 500 μM s^4U was added to each well and incubated for another 1 h.

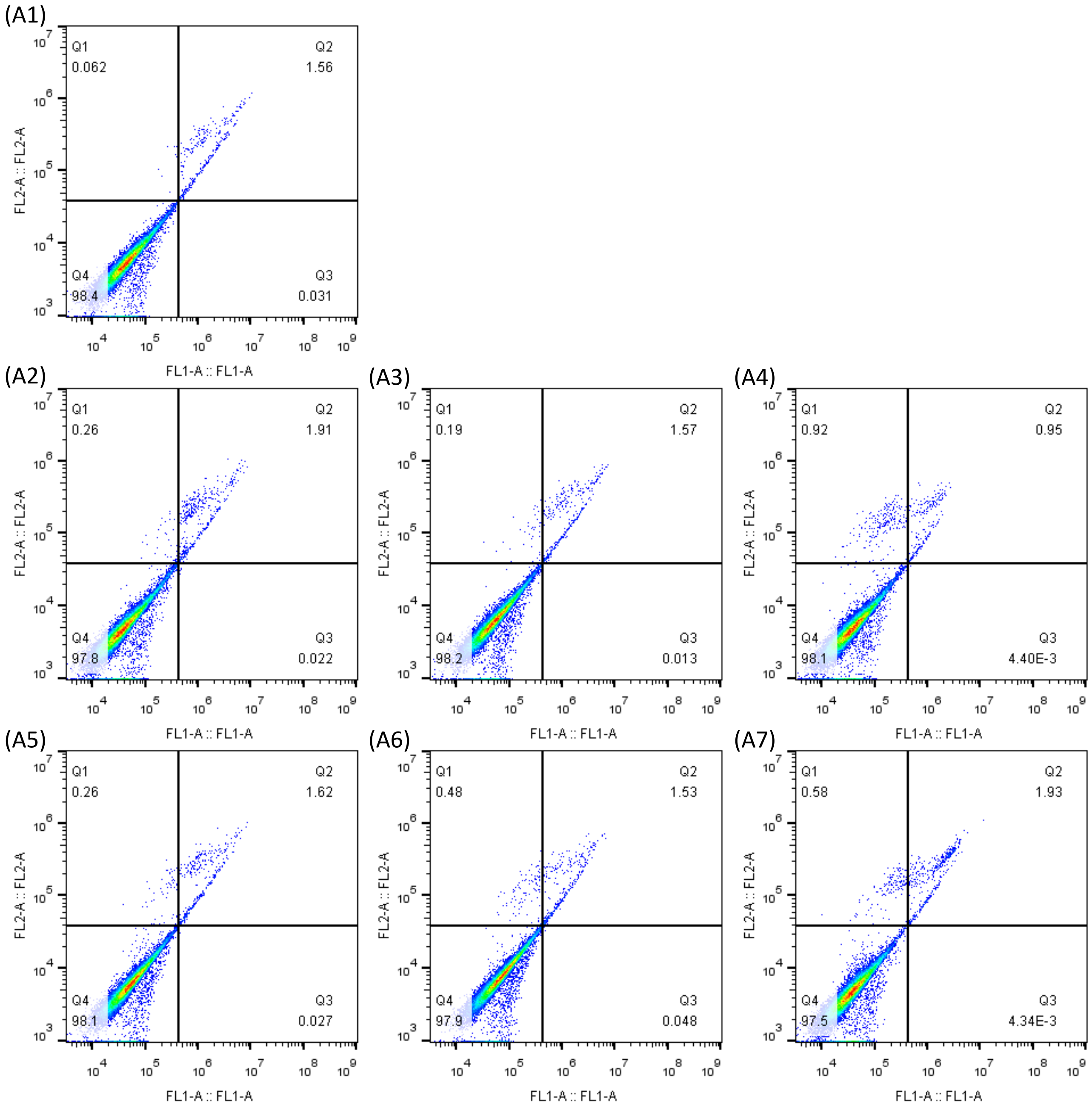

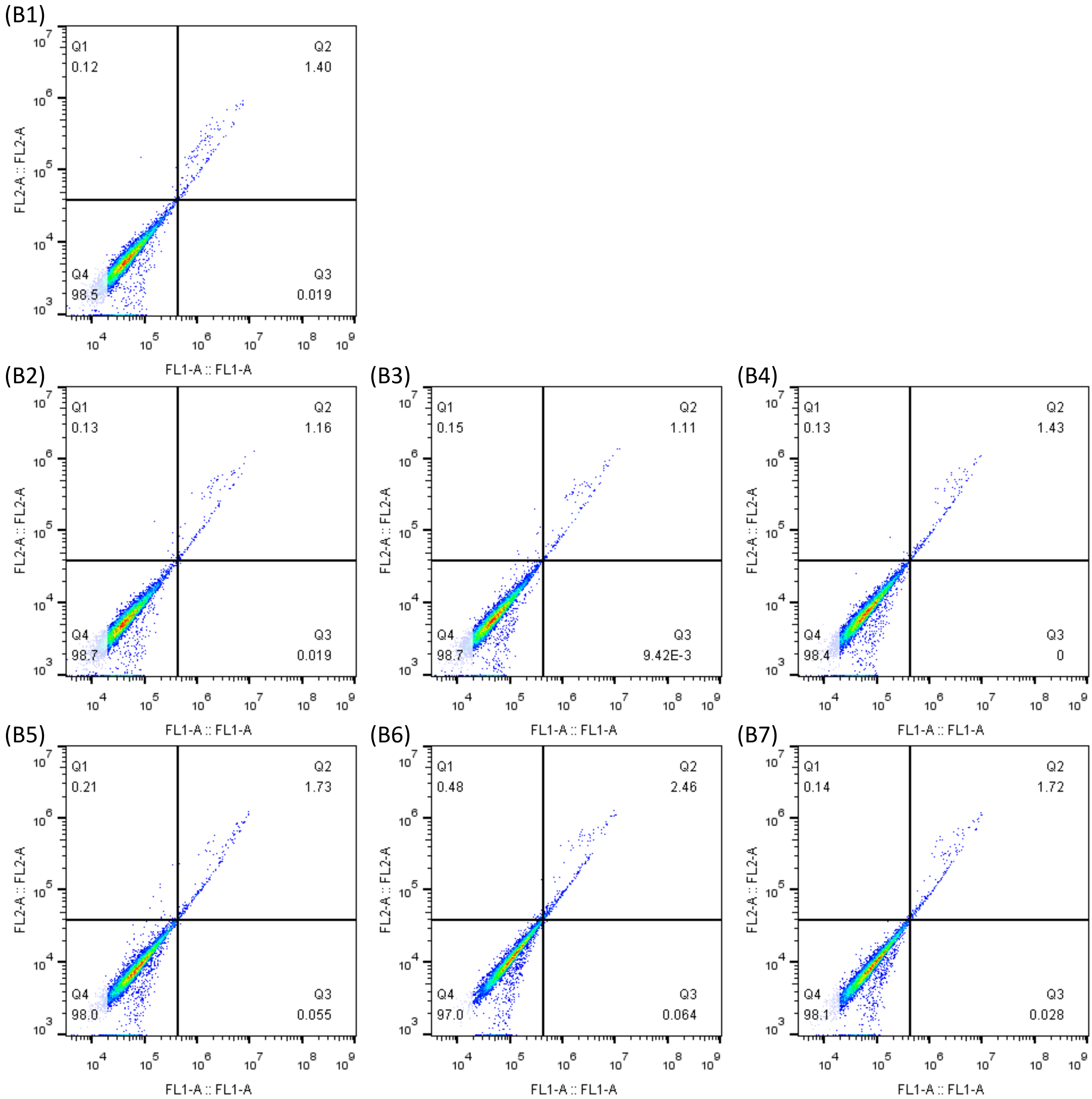
(B1)
Q1 0.12
Q2 1.40
Q4 98.5
Q3 0.019
(B2)
Q1 0.13
Q2 1.16
Q4 98.7
Q3 0.019
(B3)
Q1 0.15
Q2 1.11
Q4 98.7
Q3 9.42E-3
(B4)
Q1 0.13
Q2 1.43
Q4 98.4
Q3 0
(B5)
Q1 0.21
Q2 1.73
Q4 98.0
Q3 0.055
(B6)
Q1 0.48
Q2 2.46
Q4 97.0
Q3 0.064
(B7)
Q1 0.14
Q2 1.72
Q4 98.1
Q3 0.028
FL2-A :: FL2-A
FL1-A :: FL1-A

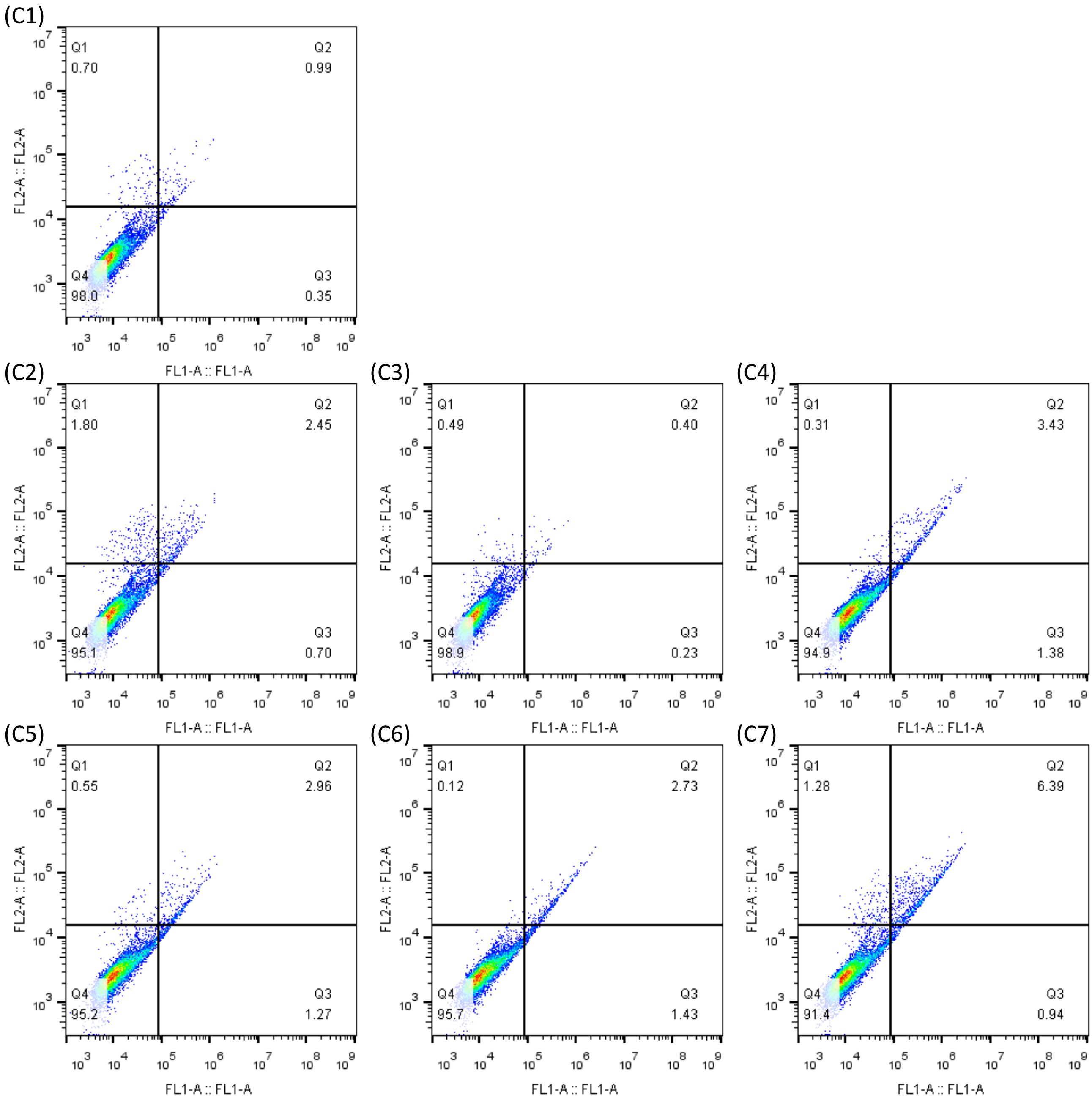

Figure S10. Apoptosis analysis in different incubation conditions, (A) cultured with increasing concentrations of PDP (1-7 stand for concentration of 0, 2, 4, 6, 8, 10 and 15 μM) for 1.5 h. After cells

incubated with PDP for 0.5 h, 500 µM s^4U was added to each well and incubated for another 1 h; (B) cultured with increasing concentrations of PDP (1-7 stand for concentration of 0, 2, 4, 6, 8, 10 and 15 µM) for 1.5 h; (C) cultured with increasing concentrations of s^4U (1-7 stand for concentration of 0, 250, 500, 1000, 2000, 5000 and 10000 µM) for 1 h.

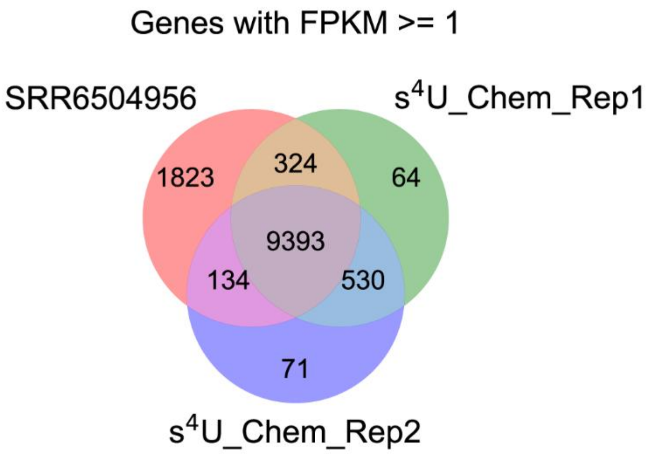

Figure S11. Venn diagram of the duplication of expressed genes in three HEK293T datasets of SRR6504956 (preparing sequencing library directly after mRNA extraction), s^4U_Chem_Rep1 and s^4U_Chem_Rep2 (incubating with s^4U and reacting with acrylonitrile before sequencing library preparation)

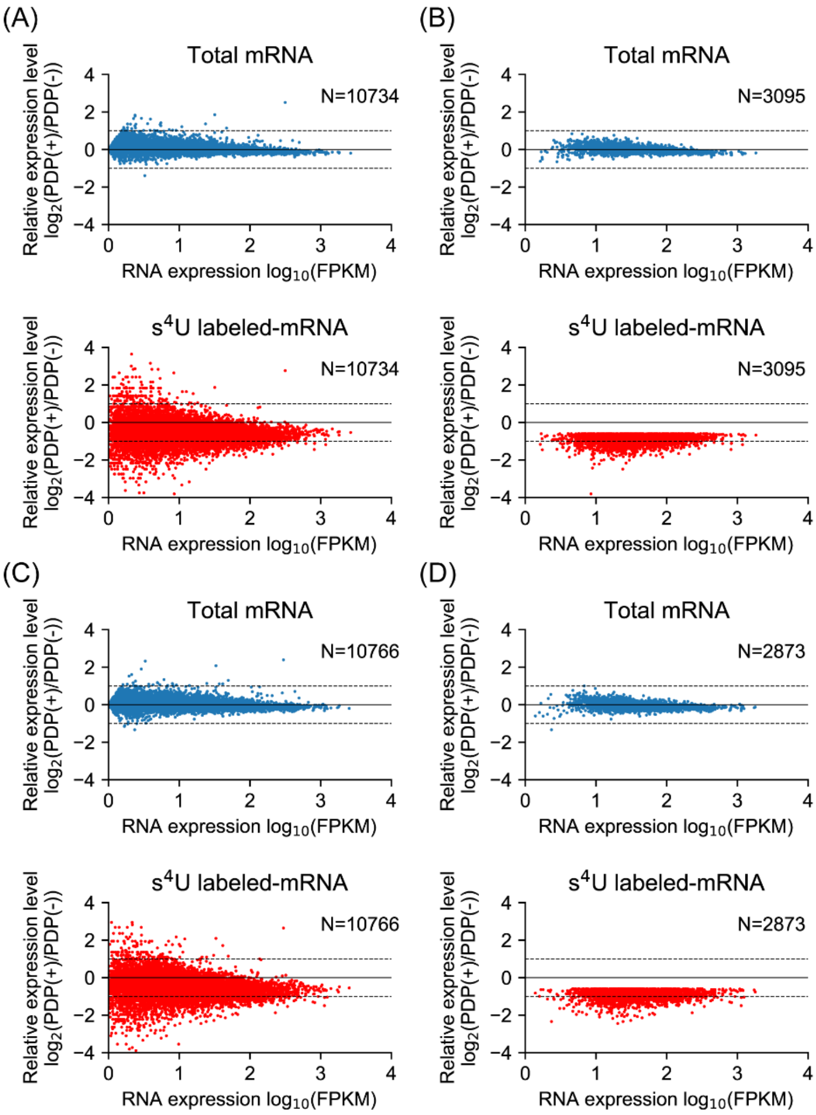

Figure S12. Global changes in the abundance of total and nascent mRNAs in HEK293T cells. (A, B) Replication No. 1; (C, D) Replication No. 2; (A, C) Abundance changes of all transcribed mRNAs in total transcripts or in nascent transcripts; (B, D) Abundance changes of total or nascent mRNAs which are downregulated in nascent transcripts. PDP(-)/PDP(+): 0/8 μM PDP treatment for 80 min.

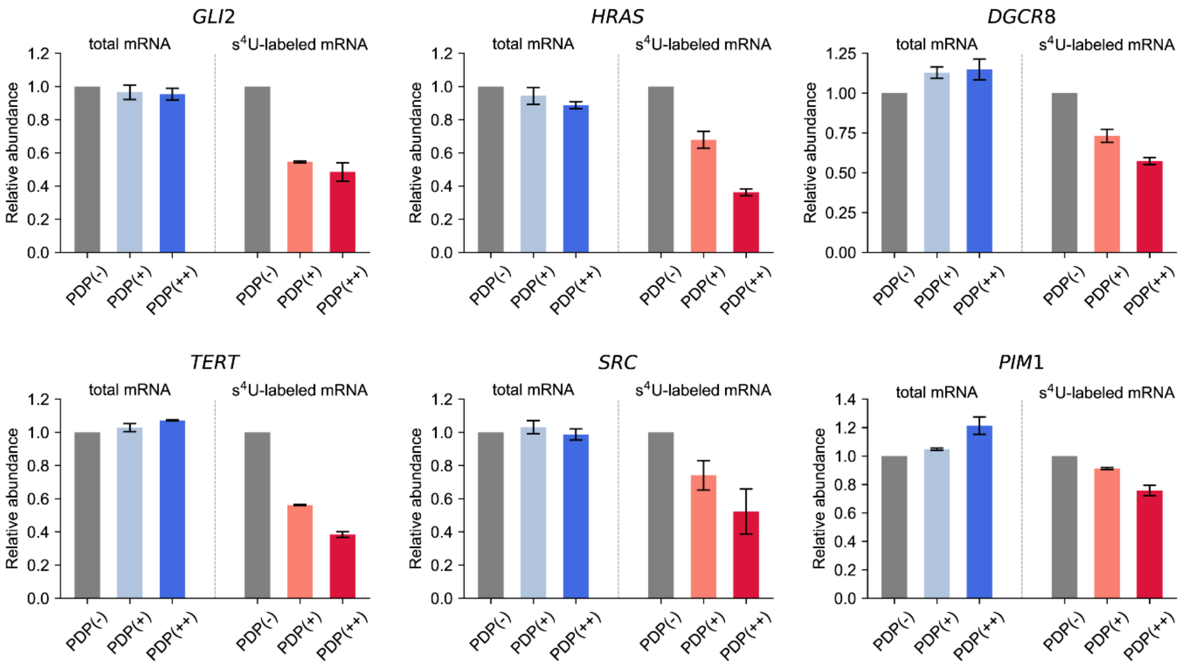

Figure S13. Comparison of the changes in the abundance of total and nascent mRNAs of six selected oncogenes in HEK293T cells. PDP(-)/PDP(+)/PDP(++): 0/4/8 μM PDP treatment for 80 min. Error bars indicate mean ± SD of two independent measurements (Sequencing).

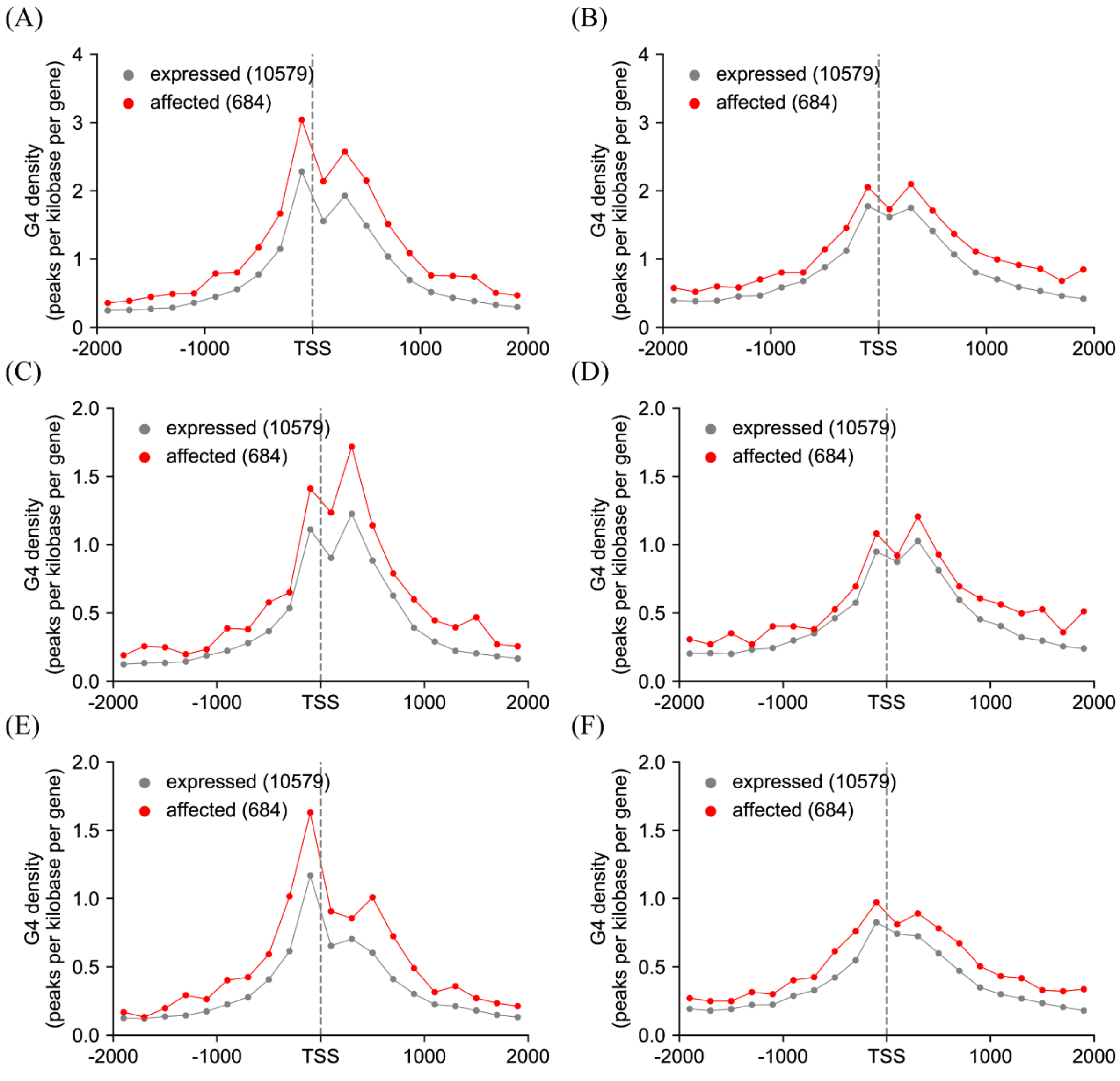

Figure S14. Comparison of G4 density at the TSS junction, in which strand discrimination has been considered, genes were divided into two categories based on changes in expression after PDP treatment in HEK293T cells, G4 sites were redicted based on G-quadruplex search algorithm $G_{3+}N_{1-7}G_{3+}N_{1-7}G_{3+}N_{1-7}G_{3+}$ [4] (A, C, E) or derived from G4-seq [5] (B, D, F). (A, B) all; (C, D) non-template strand; (E, F) template strand.

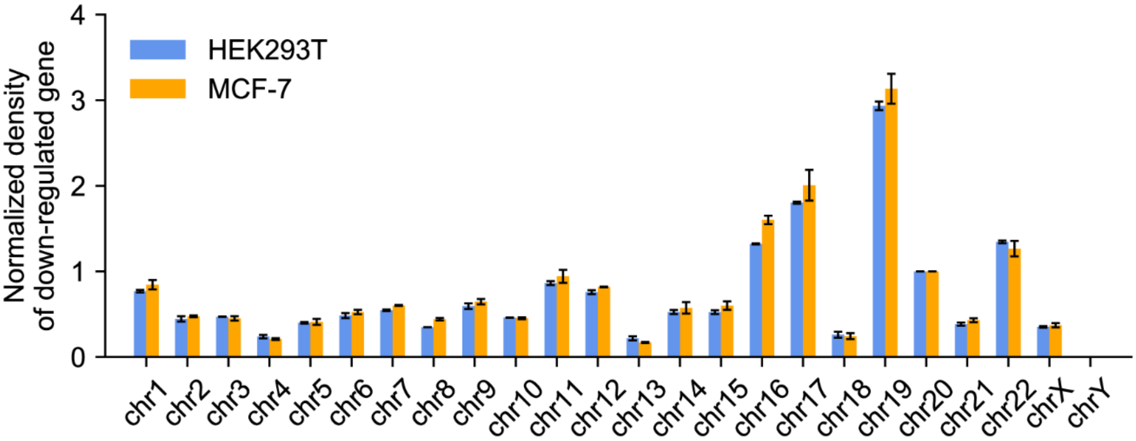

Figure S15. The frequency distribution of down-regulated genes on chromosomes after PDP (4 μM) treatment. Data was obtained by normalizing the experimental number of down-regulated genes to the size of each chromosome and then to chromosome 20. The genes whose expression levels decreased to more than 10% compared to the PDP-untreated controls were taken into account. Chromosomes 16, 17 and 19 were identified to exhibit higher PQS density, thus the down-regulated gene displayed a distribution tendency toward Chromosomes 16, 17 and 19. Error bars indicate mean ± SD of two independent measurements.

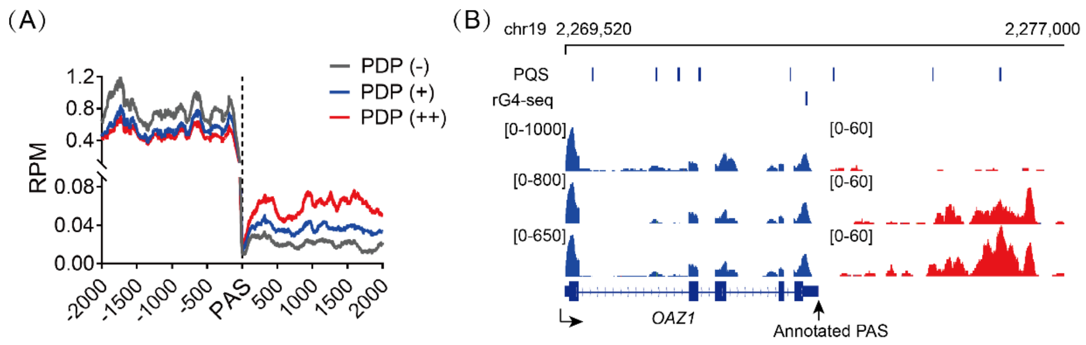

Figure S16. Discovery of the regulatory functions of PDP in pre-mRNA polyadenylation by AMUC-seq. (A) Comparison of the expression levels of transcripts around the annotated PASs between PDP-treated and PDP-untreated RNA samples; (B) Genome browser view of a representative region displays the expression pattern around PAS.

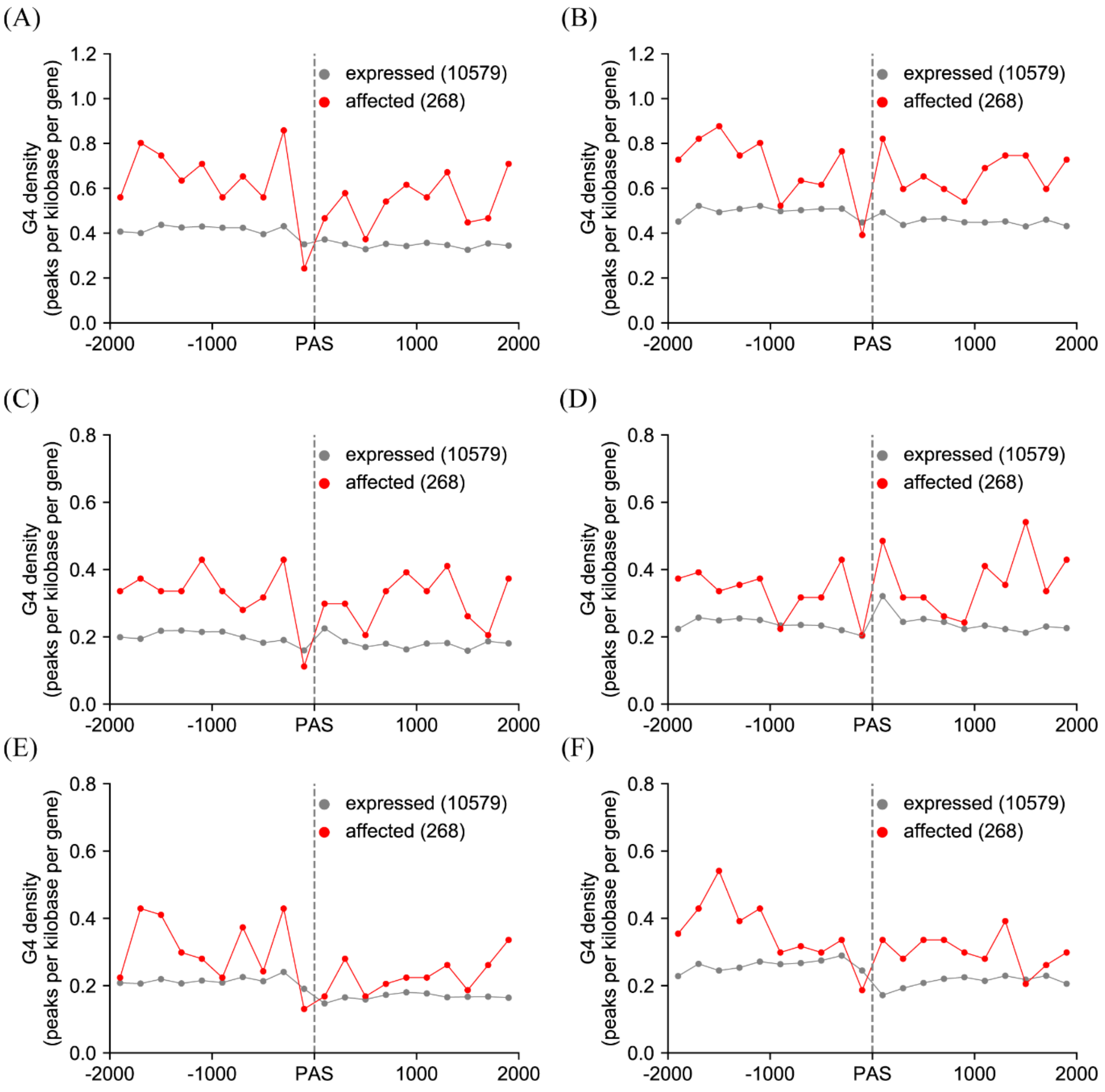

Figure S17. Comparison of G4 density around the annotated PASs between all detected expressed genes and genes with affected 3' end processing processes in HEK283T cells, G4 sites were redicted based on G-quadruplex search algorithm $G_{3+}N_{1-7}G_{3+}N_{1-7}G_{3+}N_{1-7}G_{3+}$ [4] (A, C, E) or derived from G4-seq [5] (B, D, F). (A, B) all; (C, D) non-template strand; (E, F) template strand.

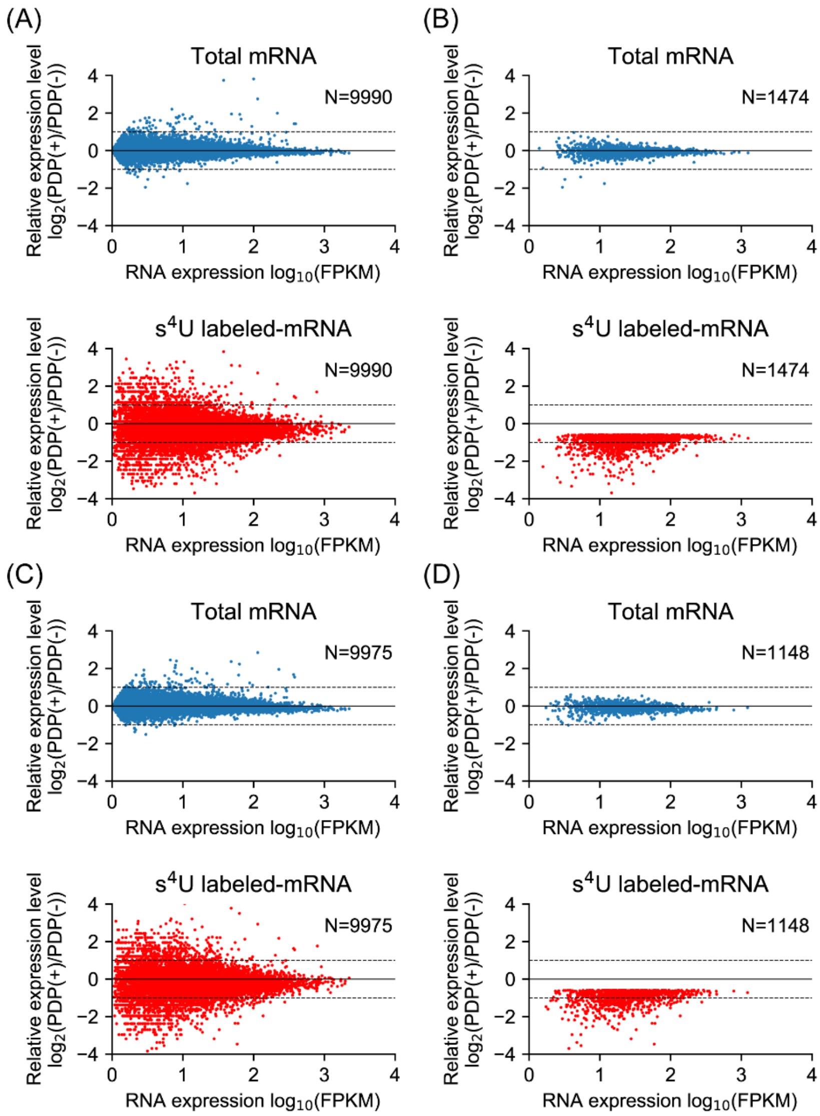

Figure S18. Global changes in the abundance of total and nascent mRNAs in MCF-7 cells. (A, B) Replication No. 1; (C, D) Replication No. 2; (A, C) Abundance changes of all transcribed mRNAs in total

transcripts or in nascent transcripts; (B, D) Abundance changes of total or nascent mRNAs which are downregulated in nascent transcripts. PDP(-)/PDP(+): 0/8 µM PDP treatment for 80 min.

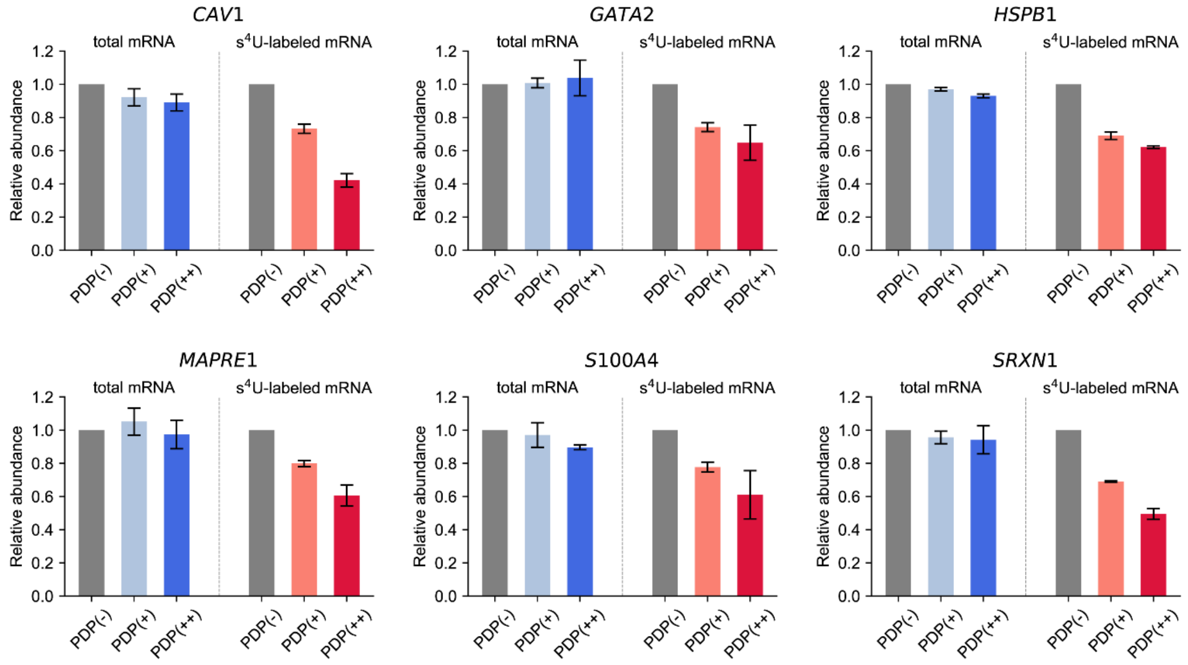

Figure S19. Comparison of the changes in the abundance of total and nascent mRNAs of six selected oncogenes in MCF-7 cells. PDP(-)/PDP(+)/PDP(++): 0/4/8 µM PDP treatment for 80 min. Error bars indicate mean ± SD of two independent measurements (Sequencing).

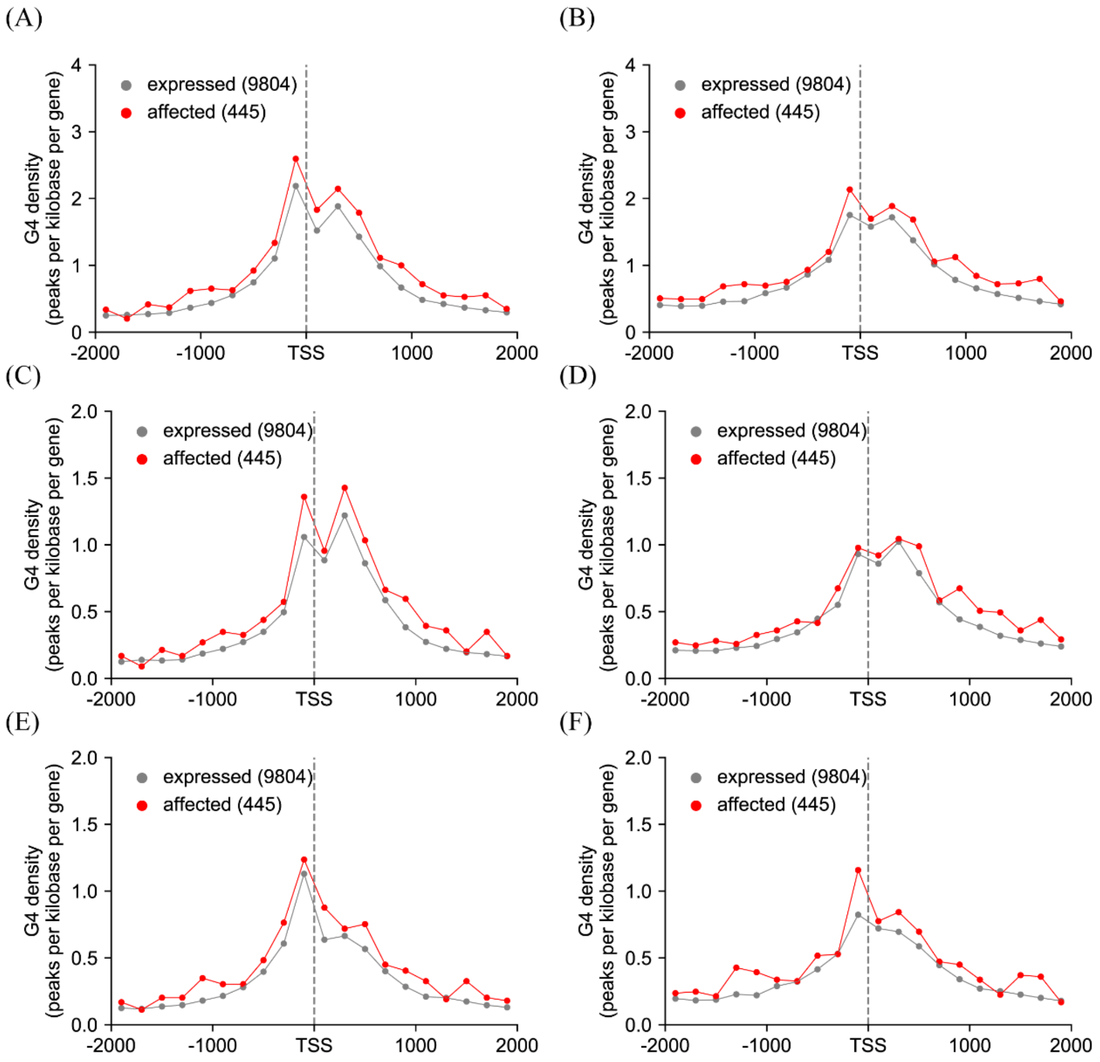

Figure S20. Comparison of G4 density at the TSS junction, in which strand discrimination has been considered, genes were divided into two categories based on changes in expression after PDP treatment in MCF-7 cells, G4 sites were redicted based on G-quadruplex search algorithm $G_{3+}N_{1-7}G_{3+}N_{1-7}G_{3+}N_{1-7}G_{3+}$ [4] (A, C, E) or derived from G4-seq [5] (B, D, F). (A, B) all; (C, D) non-template strand; (E, F) template strand.

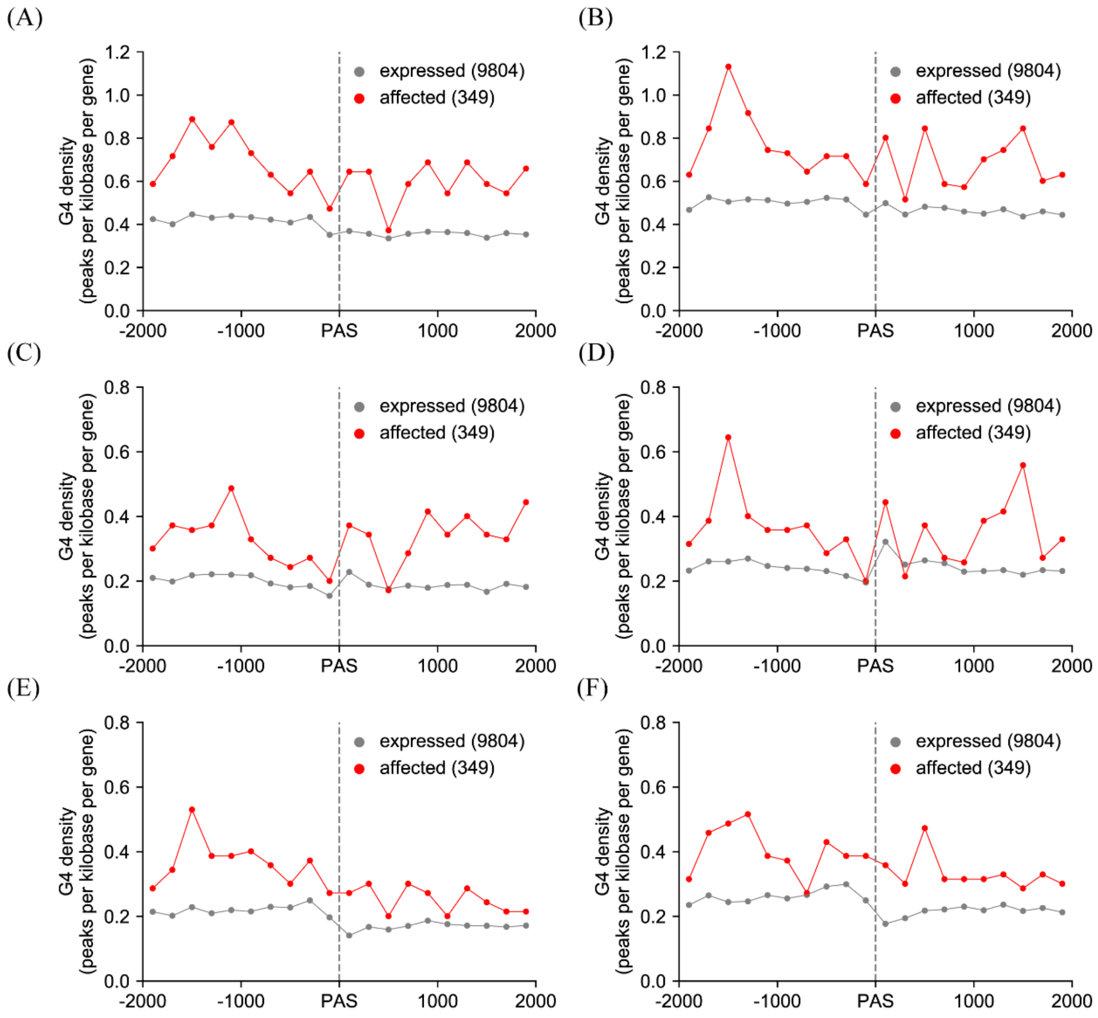

Figure S21. Comparison of G4 density around the annotated PASs between all detected expressed genes and genes with affected 3' end processing processes in MCF-7 cells, G4 sites were redicted based on G-quadruplex search algorithm $G_{3+}N_{1-7}G_{3+}N_{1-7}G_{3+}N_{1-7}G_{3+}$ [(4)] (A, C, E) or derived from G4-seq [(5)] (B, D, F). (A, B) all; (C, D) non-template strand; (E, F) template strand.

REFERENCES

1. Riml, C., Amort, T., Rieder, D., Gasser, C., Lusser, A. and Micura, R. (2017) Osmium-Mediated Transformation of 4-Thiouridine to Cytidine as Key To Study RNA Dynamics by Sequencing. *Angew. Chem. Int. Ed.*, 56, 13479-13483.

2. J. A. Schofield, E. E. Duffy, L. Kiefer, M. C. Sullivan, M. D. Simon, *Nat. Methods* **2018**, *15*, 221.

3. V. A. Herzog, B. Reichholf, T. Neumann, P. Rescheneder, P. Bhat, T. R. Burkard, W. Wlotzka, A. von Haeseler, J. Zuber, S. L. Ameres, *Nat. Methods* **2017**, *14*, 1198.

4. Huppert, J.L. and Balasubramanian, S. (2007) G-quadruplexes in promoters throughout the human genome. *Nucleic Acids Res.*, 35, 406-413.

5. Chambers, V.S., Marsico, G., Boutell, J.M., Di Antonio, M., Smith, G.P. and Balasubramanian, S. (2015) High-throughput sequencing of DNA G-quadruplex structures in the human genome. *Nat. Biotechnol.*, 33, 877-881.
